# Stemness properties of SSEA-4+ subpopulation isolated from heterogenous Wharton’s jelly mesenchymal stem/stromal cells

**DOI:** 10.3389/fcell.2024.1227034

**Published:** 2024-02-22

**Authors:** Agnieszka Smolinska, Magdalena Chodkowska, Agata Kominek, Jakub Janiec, Katarzyna Piwocka, Dorota Sulejczak, Anna Sarnowska

**Affiliations:** ^1^ Translational Platform for Regenerative Medicine, Mossakowski Medical Research Institute, Polish Academy of Sciences, Warsaw, Poland; ^2^ Laboratory of Cytometry, Nencki Institute of Experimental Biology, Polish Academy of Sciences, Warsaw, Poland; ^3^ Department of Experimental Pharmacology, Mossakowski Medical Research Institute, Polish Academy of Sciences, Warsaw, Poland

**Keywords:** mesenchymal stem/stromal cells, SSEA-4, stemness, pluripotent, FACS, MACs, heterogeneity

## Abstract

**Background:** High heterogeneity of mesenchymal stem/stromal cells (MSCs) due to different degrees of differentiation of cell subpopulations poses a considerable challenge in preclinical studies. The cells at a pluripotent-like stage represent a stem cell population of interest for many researchers worldwide, which is worthy of identification, isolation, and functional characterization. In the current study, we asked whether Wharton’s jelly-derived MSCs (WJ-MSCs) which express stage-specific embryonic antigen-4 (SSEA-4) can be considered as a pluripotent-like stem cell population.

**Methods:** SSEA-4 expression in different culture conditions was compared and the efficiency of two cell separation methods were assessed: Magnetic Activated Cell Sorting (MACS) and Fluorescence Activated Cell Sorting (FACS). After isolation, SSEA-4+ cells were analyzed for the following parameters: the maintenance of the SSEA-4 antigen expression after cell sorting, stem cell-related gene expression, proliferation potential, clonogenicity, secretome profiling, and the ability to form spheres under 3D culture conditions.

**Results:** FACS allowed for the enrichment of SSEA-4+ cell content in the population that lasted for six passages after sorting. Despite the elevated expression of stemness-related genes, SSEA-4+ cells neither differed in their proliferation and clonogenicity potential from initial and negative populations nor exhibited pluripotent differentiation repertoire. SSEA-4+ cells were observed to form smaller spheroids and exhibited increased survival under 3D conditions.

**Conclusion:** Despite the transient expression of stemness-related genes, our findings could not fully confirm the undifferentiated pluripotent-like nature of the SSEA-4+ WJ-MSC population cultured *in vitro*.

## 1 Introduction

According to the guidelines published in 2006 by the International Society for Cell & Gene Therapy, mesenchymal stem/stromal cells (MSCs) are multipotent adult cells that differentiate toward mesodermal lineage tissues: osteocytes, chondrocytes, and adipocytes (Dominici et al., 2006). However, many research groups suggested a wider MSC differentiation potential by providing protocols to obtain other cells such as neurons or hepatocytes (Zhao et al., 2016). Some researchers went even further and claimed that MSCs might manifest the properties of pluripotent-like cells by the expression of stemness-related transcription factors (such as Sox2, Nanog, and Oct4) and differentiation toward cells from all three germ layers. Even though the reported observations are controversial and disputable, the observed discrepancies between research groups can be explained by MSC heterogeneity.

Heterogeneity poses a serious issue for further research as only a small fraction of cells in MSC populations appear to fulfill functional criteria for stem cells (Ivanovic, 2023). The existence of surface antigens associated with other cell types is one of the observed aspects of MSC heterogeneity. Researchers propose numerous candidates for a genuine stem population to improve the efficiency of MSC therapies (Lv et al., 2014) based on induced pluripotent stem (iPS) and embryonic stem cell studies. Cells positive for SSEA-3, an early embryonic antigen, were confirmed to differentiate toward cells from all three germ layers (Kuroda et al., 2010) although they were present in the initial population in a negligible percentage, which does not correspond to the plasticity of MSCs. MSCs positive for CD271, an antigen typical of neural crest-derived cells, proliferated more rapidly and contained more cells capable of forming colonies (Mikami et al., 2011; Barilani et al., 2018). MSCs expressing CD146, an antigen associated with endothelial cells, exhibited a greater ability to migrate to damaged tissue (Wangler et al., 2019). CD133, a surface antigen associated with glioblastoma cells, was also suggested as a potential marker of stemness population in umbilical cord-derived MSCs (UC-MSCs) and MSCs derived from adipose tissue (AD-MSCs) (Doshmanziari et al., 2021). Another iPS- and embryonic stem cell (ESC)-expressed marker found in a much higher proportion of MSCs is stage-specific embryonic antigen-4 (SSEA-4).

SSEA-4 appears during early embryonic development (Henderson et al., 2002) but is also found on undifferentiated cells such as embryonic stem cells (ESCs) (Draper et al., 2002; Kallas et al., 2011), induced pluripotent stem cells (iPSCs) (Ojima et al., 2015), and various types of tumor cells (Sivasubramaniyan et al., 2015; Nakamura et al., 2019; Lee et al., 2021). Many publications reported the expression of SSEA-4 within MSC populations within the range of 30%–90% (Drela et al., 2016; Musiał-Wysocka et al., 2019). Despite the abundance of evidence on pluripotent-like properties of SSEA-3, researchers debate whether SSEA-4 may also be a prognostic marker for genuine stem cell populations (Gang et al., 2007; Kawanabe et al., 2012). Targeting SSEA-4 is a strategy for stem population selection in undifferentiated ESCs from differentiated derivatives (Fong et al., 2009) and neural stem/progenitor cells from the human embryonic forebrain (Barraud et al., 2007). SSEA-4 was also suggested as an identifier of tumor-initiating subpopulations and proposed as a target for the therapies (He and Garcia, 2004; Sivasubramaniyan et al., 2015; Soliman et al., 2020). In our previous paper, long-term 3D culture was observed to increase the content of SSEA-4+ cells, thereby suggesting that SSEA-4 could help toward survival under harsh 3D conditions (Kaminska et al., 2021).

This study was carried out to determine the therapeutic benefits of the SSEA-4-positive cells from Wharton’s jelly MSCs (WJ-MSCs) as a potential pluripotent-like stem cell population responsible for so-called “MSCs plasticity” with restorative (replacing injured cells) properties. To identify the MSC-SSEA-4 + subpopulation’s unique properties, it was compared both to the negative population, without SSEA-4, (WJ-MSC-SSEA-4-) and to the heterogenous MSC populations (unsorted WJ-MSC) (experimental steps explained in Supplementary Figure S1). Our experiments allowed us to establish the most favorable conditions for SSEA-4 expression and separation while the positive subpopulation analyses provided full characteristics of stemness-related properties.

## 2 Materials and methods

### 2.1 WJ-MSCs isolation and primary culture

Human umbilical cords were acquired from full-term deliveries with the written consent of the mother according to the Warsaw Medical University Ethics Committee Guidelines (KB/213/2016). The cords (15–20 cm long) were first transported in phosphate-buffer saline solution (PBS; Sigma-Aldrich, Saint Louis, MO, United States) with a mixture of penicillin-streptomycin-amphotericin B (1:100, Gibco, Thermo Fisher Scientific, Walthman, United States) and then cut into slices with a lancet (slice thickness: 2–3 mm). Wharton’s jelly cylindrical fragments of 2–3 mm in diameter were obtained from the umbilical cord using the diameter biopsy punch (Miltex, GmbH, Viernheim, Germany). The explants were transferred to 6-well culture plates and cultured in the following medium standard for WJ-MSC culture: DMEM (Gibco), 5% human platelet cell lysate (Mill Creek Life Sciences, Rochester, MN, United States), and penicillin-streptomycin-amphotericin B (1:100; Gibco). The following cell culture conditions were applied: adherent surface, 37^O^C temperature, 95% humidity, 5% CO_2_ concentration, and 5% O_2_ concentration. The culture medium was replaced every 2 days for 14 days *in vitro*. The cells were cultured until they migrated out of the explant and the culture reached semiconfluence; then the cells were detached with Accutase Cell Detachment Solution (Beckton Dickinson, Franklin Lakes, NJ, United States) and counted.

We compared SSEA-4 expression in populations cultured with different human platelet cell lysates such as PLTGold Clinical Grade (Mill Creek Life Sciences), MultiPL’30 (Macopharma), and MultiPL’100 (Macopharma, Tourcoing, France). In further experiments, PLT Gold Clinical Grade was used as a lysate. The WJ-MSCs were cultured under the conditions described above for three passages. The cells were collected and cell sorting was performed.

### 2.2 Flow cytometry

The cells were detached with Accutase Cell Detachment Solution (BD), washed in PBS, and resuspended in Stain Buffer (BD). Flow cytometry analyses were performed with antibodies listed in Table 1. The cells were incubated in diluted antibodies in the dark for 30 min. After incubation, the cells were washed twice with Stain Buffer (BD) and resuspended in Stain Buffer. The resuspended cells were analyzed using FACS Canto II (BD) with FACSDiva software (BD) and FlowJo 10 (BD). The following laser configurations were applied: violet - 407 nm (detectors: 510/50, 450/50), blue - 488 nm (detectors: 488,10, 530/30, 585/42, 670LP, 780/60), and red - 633 nm (detectors: 660/20, 780/60). The gating strategy was presented in online resources (Supplementary Figure S2).

**TABLE 1:** List of antibodies used in flow cytometry analysis.

Specificity	Fluorochrome	Isotype	Company	Catalog number
SSEA-4	PerCP-Cy5.5	Mouse IgG3 κ	BD	561565
Isotype control	PerCP-Cy5.5	Mouse IgG3 κ	BD	561572
CD271	PE	Mouse IgG1 κ	BD	560927
CD146	PE	Mouse IgG1 κ	BD	550315
Isotype control	PE	Mouse IgG1 κ	BD	555749
CD133	Brilliant Violet 421	Mouse IgG2B κ	BD	566598
Isotype control	Brilliant Violet 421	Mouse IgG2B κ	BD	562748
CD49F	PE	Rat IgG2A κ	ThermoFischer Scientific	12-0495-81
Isotype control	PE	Rat IgG2A κ	ThermoFischer Scientific	12-4321-80

### 2.3 AD-MSCs culture

AD-MSCs isolation and culture were accepted by the Bioethical Committee at the Centre of Postgraduate Medical Education (No. 63/PB/2013) on 25 September 2013, according to the guidelines of the Declaration of Helsinki. Adipose tissue was collected during liposuction in the Plastic Surgery Department at Orlowski’s Clinical Hospital in Warsaw. The AD-MSCs were isolated according to the previously described protocol (Figiel-Dabrowska et al., 2021; Rybkowska et al., 2023). The isolated AD-MSCs were in MEM α (Gibco), 5% human platelet lysate (Mill Creek Life Sciences), and 1% penicillin-streptomycin-amphotericin B (1:100; Gibco). The following cell culture conditions were applied: adherent surface, 37^O^C temperature, 95% humidity, 5% CO_2_ concentration, and 5% O_2_ concentration. The culture medium was changed every 2–3 days and AD-MSCs were passaged when the culture reached semiconfluence. AD-MSCs from the third passage were detached with Accutase Cell Detachment Solution and washed with PBS twice. AD-MSCs were used for flow cytometry and prepared in the manner described above.

### 2.4 Magnetic activated cell sorting (MACS) isolation of SSEA-4+ cells

The WJ-MSCs from the third passage were used for MACS separation of WJ-MSC-SSEA-4+ cells. WJ-MSCs were detached and counted. The collected cells (∼2-10 × 10^6^) were incubated with magnetic beads using an anti-SSEA-4 MicroBead kit for 20 min in the dark. Then, the cells were washed with PBS. The cells were resuspended in PBS with 1% BSA and loaded into the autoMACS Pro Separator (Miltenyi Biotec, Bergisch Gladbach, Germany). The cells were run through the magnetic field with the Possel S. For further analysis, we used a positive cell population retained within the column and eluted as the second fraction was collected. After MACS sorting, the cells were counted with Trypan Blue on a hemocytometer to calculate the total cell number and cell viability. Then, the cells were subcultured for further experiments as described above.

### 2.5 Fluorescence-activated cell sorting (FACS) isolation of SSEA-4+ cells

The WJ-MSCs from the third passage were used for FACS separation of WJ-MSC-SSEA-4+ cells. The cells were stained as was the case in flow cytometry staining described in Section 2.2. After incubation and washing, the cells were sorted using FACS Aria IIu (BD) in the Laboratory of Cytometry, Nencki Institute of Experimental Biology, Warsaw. The following laser configurations were applied: violet - 407 nm (detectors: 450/40, 530/30), blue - 488 nm (detectors: 488/10, 530/30, 585/42, 616/23, 695/40, 780/60), and red - 633 nm (detectors: 660/20, 780/60). The gating strategy is presented in online resources (Supplementary Figure S3). The cells were collected from both populations, SSEA-4- and SSEA-4+, and resuspended in the cell culture medium. Directly after FACS sorting, the obtained population was analyzed with FACS Aria again to confirm the purity of sorting. Then, the cells were transported to our laboratory where we counted the total cell number and viability with Trypan Blue on a hemocytometer. Then, the cells were seeded in a culture dish and subcultured for further experiments as described above.

### 2.6 Parameters of cell sorting

The following parameters of MACS and FACS sorting, which are recovery, survival, and yield, were compared in order to determine a more efficient method of SSEA-4+ cell separation. Recovery was expressed as the ratio of the number of cells obtained in the positive fraction to the number of cells used in the sorting. To calculate recovery, we counted cells prior to sorting and in post-sorting fractions. To describe survival, we investigated the mortality of cells in samples received after cell separation with Trypan Blue staining. The yield was expressed as the ratio of positive cell content before and after cell separation. Purity was described as the percentage of SSEA-4+ cells received in a positive population sample. To estimate yield, the samples were analyzed with flow cytometry.

### 2.7 Immunocytochemistry

Immunocytochemistry was performed to detect SSEA-4 and CD90, one of the surface antigen characteristics of MSCs, for the following populations: unsorted WJ-MSC, WJ-MSC-SSEA-4-, and WJ-MSC-SSEA-4+. WJ-MSCs were washed with PBS and fixed in 4% PFA for 15 min. The samples were incubated with a blocking mixture consisting of 10% Goat Serum (Sigma Aldrich) and 1% bovine serum albumin (Sigma Aldrich) for 1 h at room temperature (RT). In the next step, primary antibodies were applied for 24 h at 4°C (Table 2). The next day, the cells were washed with PBS and then incubated with secondary antibodies conjugated with fluorochrome for 1 h (Table 2). Finally, the samples were mounted with Fluoromont-G with DAPI (Gibco) that stained cell nuclei. The analysis was performed using a confocal microscope (Zeiss, Oberkochen, Germany).

**TABLE 2:** List of antibodies used for immunocytochemistry.

Antigen	Isotype	Dilution	Company	Applied secondary antibody	Secondary antibody fluorochrome
SSEA-4	Mouse IgG3	1:200	Merck	Goat anti-IgG3	Alexa Fluor 488
CD90	Mouse IgG1	1:200	Santa Cruz	Goat anti-IgG1	Alexa Fluor 546
SOX17	Goat IgG H + L	1:100	R&D	Donkey anti-IgG	Alexa Fluor 488
Otx2	Goat IgG H + L	1:100	R&D	Donkey anti-IgG	Alexa Fluor 488
Brachyury	Goat IgG H + L	1:100	R&D	Donkey anti-IgG	Alexa Fluor 488

### 2.8 Real time-quantitative polymerase chain reaction (RT-qPCR)

Total RNA was isolated from the following groups: unsorted WJ-MSCs, negative and positive populations after FACS sorting (WJ-MSC-SSEA-4- p0 and WJ-MSC-SSEA4+ p0, respectively), and negative and positive fractions cultured for 1 passage *in vitro* (WJ-MSC-SSEA-4- p1 and WJ-MSC-SSEA4+ p1, respectively). RNA isolation was performed using the following kits depending on the cell number: Total RNA Mini Plus kit (A&A Biotechnology, Gdynia, Poland) and Total RNA Mini Plus Concentrator (A&A Biotechnology) according to the manufacturer’s protocols. After isolation, the RNA was eluted with 20 µL of RNase-free H_2_O (Sigma Aldrich). The quantity and quality of RNA were assessed using a NanoDrop 2000 spectrophotometer (Thermo Scientific). Genomic DNA (gDNA) contamination was eliminated in all RNA samples using a Clean-up RNA Concentrator (A&A Biotechnology, Thermo Fisher Scientific, Walthman, United States).

The reverse transcription process was generated using a High-Capacity RNA-to-cDNA™ Kit (Applied Biosystems) according to the manufacturer’s instructions. After receiving complementary strand DNA (cDNA), the samples were diluted in RNase-free water. Quantitative polymerase chain reactions were performed using SYBR green Master Mix (Applied Biosystems) and specific primers (Supplementary Table S1) with the 7,500 Real-Time PCR System (Applied Biosystems). The relative amount of RNA was calculated using the comparative delta-delta Ct method (2^−ΔΔCT^) and gene expression was normalized using β-actin (ACTB), while the unsorted population was used as a reference group. Gene expression was compared with the mean level of corresponding gene expression in cells of the unsorted population and expressed as an n-fold ratio. Gene expression was compared with the mean level of corresponding gene expression in cells of the unsorted population and expressed as an n-fold ratio.

### 2.9 Three germ layer differentiation potential evaluation

Three germ layer differentiation potential was determined for unsorted, positive, and negative populations. After FACS separation, the cells were seeded on a 6-well plate and cultured in a standard culture medium with the addition of basic fibroblast growth factor (bFGF) (Gibco) until they reached 70%–80% confluency. Differentiation assay was performed with the Human Pluripotent Stem Cell Functional Identification Kit (Biotechne, R&D Systems, Minneapolis, MN, United States), which is dedicated to the differentiation of iPSCs. The cells were cultured according to the manufacturer’s protocol, with a culture medium based on DMEM. After 4 days of *in vitro* culture, the cells were collected for RNA isolation and evaluation for gene expression of OTX2, Brachyury, and SOX17 (scheme of the experiment presented in Supplementary Figure S4; primers sequences can be found in Supplementary Table S1).

### 2.10 Colony forming unit (CFU) assay

To perform the CFU assay, unsorted WJ-MSCs, WJ-MSC-SSEA-4-, and WJ-MSC-SSEA-4 + were seeded on a 6-well plate, 10 cells per well. The cells were cultured for 10 days *in vitro* under standard conditions. The cells were washed with PBS, fixed with 4% PFA for 15 min, and washed with PBS again. The fixed cells were stained with 0.5% toluidine blue for 20 min and washed with distilled water after staining. The number of colonies containing 50 cells or more was counted and fibroblast colony-forming units (CFU-F) were calculated as a percentage of seeded cells.

### 2.11 Proliferation analysis

Cell proliferation was estimated as population duplication time (PDT) for four passages after cell sorting for the following populations: unsorted WJ-MSC, WJ-MSC-SSEA-4-, and WJ-MSC-SSEA-4+. The cells from each group were counted in Trypan Blue, seeded at a density of 2000 cells/cm2, and cultured under standard conditions. After 5 days of *in vitro* culture, the cells were collected, counted, and reseeded again at initial density. The PDT value was calculated according to the following equation: $PDT=\frac{\left(t-t_{0}\right)\times \log2}{logN-\log N_{0}}$ , where N is the number of cells obtained at the end of the passage, N_0_ is the initial number of the seeded cells and t-t_0_ is the duration of the passage (counted in days).

### 2.12 Soluble secretome analysis

Soluble secretome was analyzed with human Magnetic Luminex Assay (R&D Systems, Minneapolis, MN, United States) for unsorted, positive, and negative populations. For this purpose, the cells after FACS sorting were seeded at a density of 2,000 cells per cm^2^. The medium from the cell culture was collected at two time points: 3 days and 5 days *in vitro* after FACS sorting. The standard culture medium was used as a negative control. The levels of the following molecules were measured: epithelial growth factor (EGF), bFGF, glial cell line-derived neurotrophic factor (GDNF), brain-derived neurotrophic factor (BDNF), chemokine ligand 2 (CCL2), leukemia inhibitory factor (LIF), angiogenin, vascular endothelial growth factor-c (VEGF-c), and intercellular adhesion molecule 1 (ICAM-1). The actual levels of secreted factors were determined by subtraction of the negative control values from the obtained results. Luminex assay was performed according to the manufacturer’s protocol and measured in Bio-Plex 200 System (Bio-Rad Bio-Rad, Hercules, CA, United States).

### 2.13 3D culture of WJ-MSCs

Unsorted WJ-MSCs from the third passage and WJ-MSCs directly after cell sorting (WJ-MSC-SSEA-4- and WJ-MSC-SSEA-4+) were collected, counted, and seeded in antiadhesive 6-well plates (Nunclon Sphera, Thermo Fischer Scientific) at a density of 10^5^ cells per 1 mL. The cells were cultured as spheroids in culture medium and the conditions described above for 72 h *in vitro*. The diameters and numbers were measured after 24, 48, and 72 h of 3D culture. After 72 h of 3D culture, spheroids were collected and dissociated with Accutase Cell Detachment Solution (BD) for further viability analysis.

### 2.14 Viability test after 3D culture

To estimate the number of alive and dead cells after 3D culture, a viability test was performed using a mix of ethidium homodimer-1 (8 μM, EthD-1, Invitrogen) Calcein AM (Cal-AM) (0,1 μM, Invitrogen) and Hoechst 33,342 dye (1 μg/mL; Sigma Aldrich). Spheroids or single cells derived from dissociated spheroids were incubated with a staining mixture for 45 min at room temperature in darkness. The stained cells were observed in the Axio Vert.A1 fluorescence microscope (Zeiss). Dead and alive cells were calculated automatically with the ZEISS ZEN 2.0 Blue Edition software.

### 2.15 Statistics

The experiments were performed on the cells obtained from at least three WJ donors (n ≥ 3). Normality was examined with the Shapiro-Wilk normality test. The unpaired t-student test was used for the data from two groups with normal distribution. The data from multiple groups with normal distribution were analyzed by using a one-way analysis of variance (ANOVA), followed by Tuckey’s multiple comparison test. For the non-moral distribution, the data was analyzed using the Kruskal–Wallis test, followed by Dunn’s multiple comparison test. The results are presented as mean ± standard deviation (SD) for parametric tests or as median ±95% confidence interval (95% CI) for non-parametric tests. The results were considered statistically significant when the *p*-value was higher than 0.05. Statistical analysis was conducted with the GraphPad Prism 7 software.

## 3 Results

### 3.1 Expression of SSEA-4 in the heterogenous WJ-MSCs population

**FIGURE 1:**
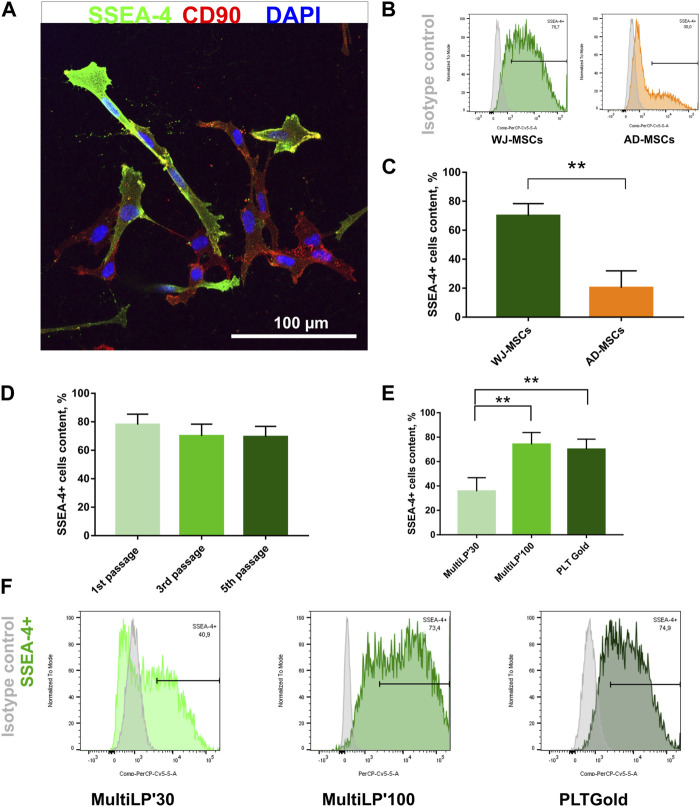
SSEA-4+ cells content in the heterogenous population of WJ-MSCs. **(A)** SSEA-4 surface antigen together with CD90 in the heterogenous WJ-MSC populations; immunocytochemical staining. The white scale bar indicates 100 µm. **(B)** Representative histograms from flow cytometry analysis for SSEA-4+ population in WJ-MSCs and AD-MSCs. **(C)** Comparison of SSEA-4+ cells in MSCs isolated from different sources: Wharton’s Jelly (WJ-MSCs) and adipose tissue (AD-MSCs). Passage number of analyzed cells: third passage; applied lysate platelet: PLTGold. **(D)** SSEA-4+ cell content in the WJ-MSCs from first, third, and fifth passage of cell culture; flow cytometry. **(E)** SSEA-4+ cell content in the WJ-MSC populations cultured with different platelet lysates: MultiLP’30 (Macopharma), MultiLP’100 (Macopharma), and PLTGold (Mill Creek Life Sciences); flow cytometry analysis. **(F)** Representative histograms from flow cytometry analysis for the SSEA-4+ population from WJ-MSCs cultured in different platelet lysates: MultiLP’30, MultiLP’100, and PLTGold. For **(B)**, **(D)**, and **(E)** results are presented as mean values of three experiments ±SD. *p*-value for ** <0.01.

WJ-MSCs used for the experiments exhibited surface antigens recommended by *The International Society for Cell & Gene Therapy* for MSC characteristics (Supplementary Figure S5; Supplementary Table S2) and differentiated toward mesodermal lineage cells: osteocytes, adipocytes, and chondrocytes (Supplementary Figure S6). All MSC-SSEA-4+ cells expressed the CD90, which is one of the recommended MSC antigens (Figure 1A; negative controls are presented in online resources: Supplementary Figure S7). To establish the most favorable conditions, SSEA-4+ cells present in heterogenous MSCs were estimated with regard to i) the source of tissue, ii) the passage number, and iii) the culture medium. SSEA-4+ cell content was compared in two MSC populations derived from different tissues: Wharton’s jelly and adipose tissue (Figures 1B,C). Nearly 3.5 times more SSEA-4+ cells were detected in WJ-MSCs than in AD-MSCs (70% ± 8.3% and 20.3% ± 11.7, respectively). No significant changes were observed in SSEA-4 expression in WJ-MSCs obtained from the first, third, and fifth passage of *in vitro* culture (Figure 1D). To choose the most optimal culture medium composition, three commercially available platelet lysates were compared as a source of proteins and tropic factors: MultiLP’30, MultiLP’100, and PLTGold Clinical Grade (Figures 1E,F). MultiLP’100 and PLT Gold lysates were more abundant in growth factors than MultiLP’30. The proportion of positive cells was the lowest under cell culture with MultiLP’30 lysate (35.7% ± 11.1), while it was found to increase in cultures using MultiLP’100 and PLTGold lysates (74% ± 9.7% and 70% ± 8.3, respectively) (Figure 1F). The application of a higher concentration of platelet lysate was also observed to increase the content of double-positive SSEA-4+ CD271+ cells in WJ-MSC populations (Supplementary Figure S8). Although almost all WJ-MSC-SSEA-4+ cultured with lysates at a higher concentration expressed CD271, a similar analysis in AD-MSC populations revealed that it was CD271+ cells that were a separate subpopulation of SSEA-4+ cells (Supplementary Figure S6D). Consequently, WJ-MSCs from the third passage, cultured with PLTGold human platelet lysate, were selected for further experiments and analysis.

### 3.2 Comparison of sorting methods for WJ-MSC-SSEA-4+ cell separation

**FIGURE 2:**
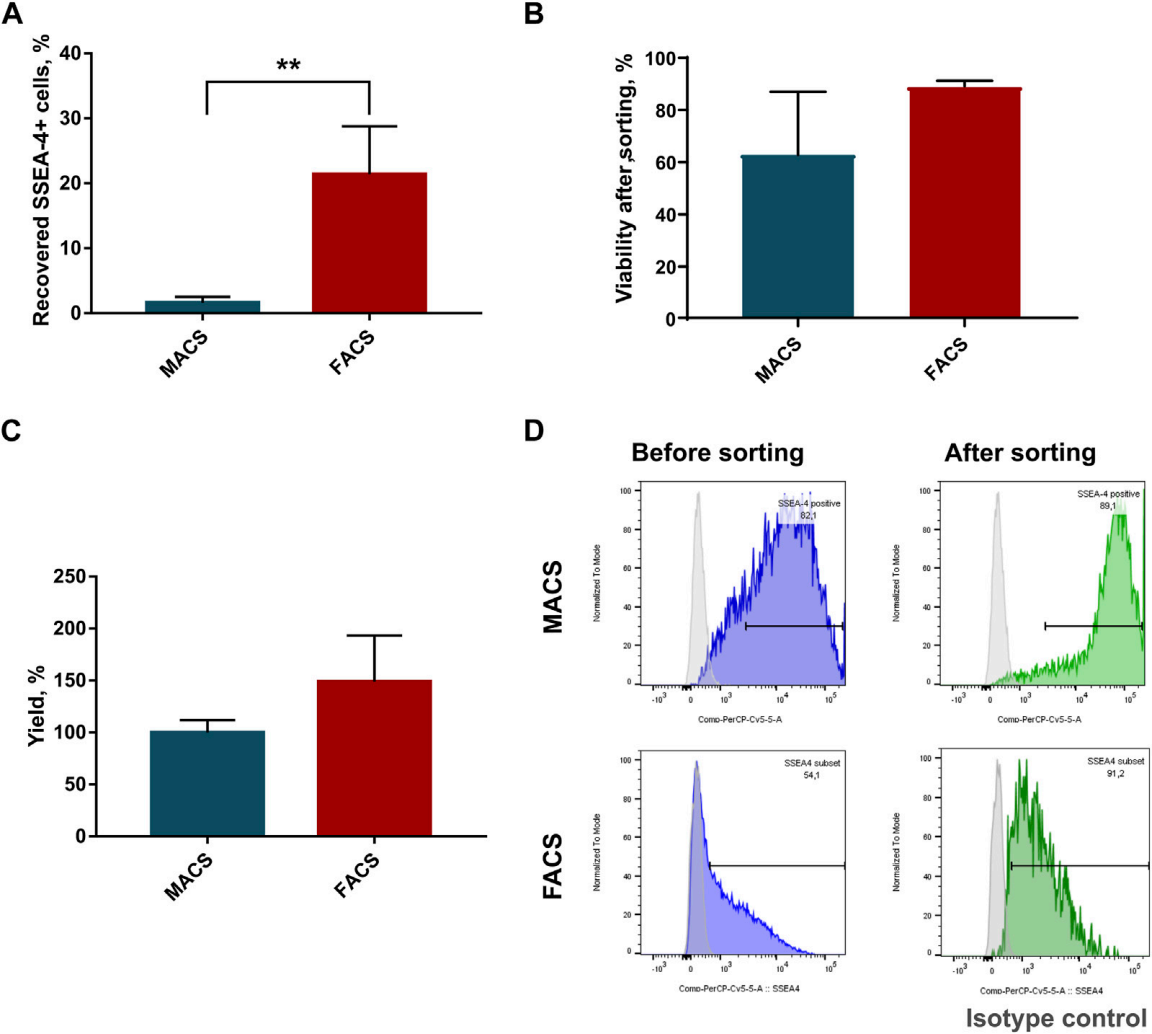
Parameter comparison of sorting methods: Magnetic Activated Cell Sorting (MACS) and Fluorescence Activated Cell Sorting (FACS). **(A)** Percent of recovered SSEA-4+ cells after cell sorting; *p*-value for **<0.01. **(B)** Viability of cells in population after cell sorting. **(C)** Sorting yield expressed as a change in SSEA-4+ cell content calculated before and after sorting. **(D)** Representative histograms from flow cytometry analyses of SSEA-4 cell content before and after sorting for MACS and FACS were used to calculate yield. For **(B)** and **(D)** results are presented as mean values of at least four experiments ±SD. *p*-value for ** <0.01.

To choose a more optimal WJ-MSC-SSEA-4+ cell separation method, MACS and FACS were considered. The following parameters were compared: recovery, which was expressed as the ratio of the positive cell numbers obtained to the cell number used in sorting; survival, which was expressed as cell mortality and yield and as the ratio of positive cells before and after sorting. FACS allowed us to obtain more than 13 times as many cells as with MACS(MACS: 1.6% ± 0.9 vs. FACS: 21.4% ± 7.4) (Figure 2A). We observed a decrease in the cell viability after MACS sorting, but the differences were not statistically significant due to substantial discrepancies in the values obtained in MACS sorting (MACS: 62.75% ± 24.3 vs. FACS: 89% ± 2) (Figure 2B). Our calculations performed with Trypan Blue staining were also supported by EthD-1 staining used in the pilot experiment (Supplementary Figure S9). A slightly higher yield was recorded for the FACS method, with the differences being statistically insignificant (MACS: 99.9% ± 11.9 vs. FACS: 148.9% ± 44.4) (Figure 2C). SSEA-4+ cell content recorded before and after sorting was compared to calculate yield value (Figure 2D). Overall, FACS sorting was selected for our further experiments as the method showed superior cell recovery and tendencies toward decreased mortality and increased yield.

Figure 2D shows a significant difference in SSEA4 expression in the initial population, which resulted from the difference in the cytometer assigned to the method. MACS sorting was analyzed using FACS Canto. For FACS sorting, we used the integrated FACS Aria IIu (BD), which differed in the detector settings (as described in the materials and method section). The results obtained may explain the discrepancies in the SSEA4 expression of the different study groups. Nevertheless, the same cytometer was always used in our further experiments, such as the persistence of SSEA-4+ cells after sorting or co-expression of other surface antigens.

### 3.3 SSEA-4+ cells after FACS separation

**FIGURE 3:**
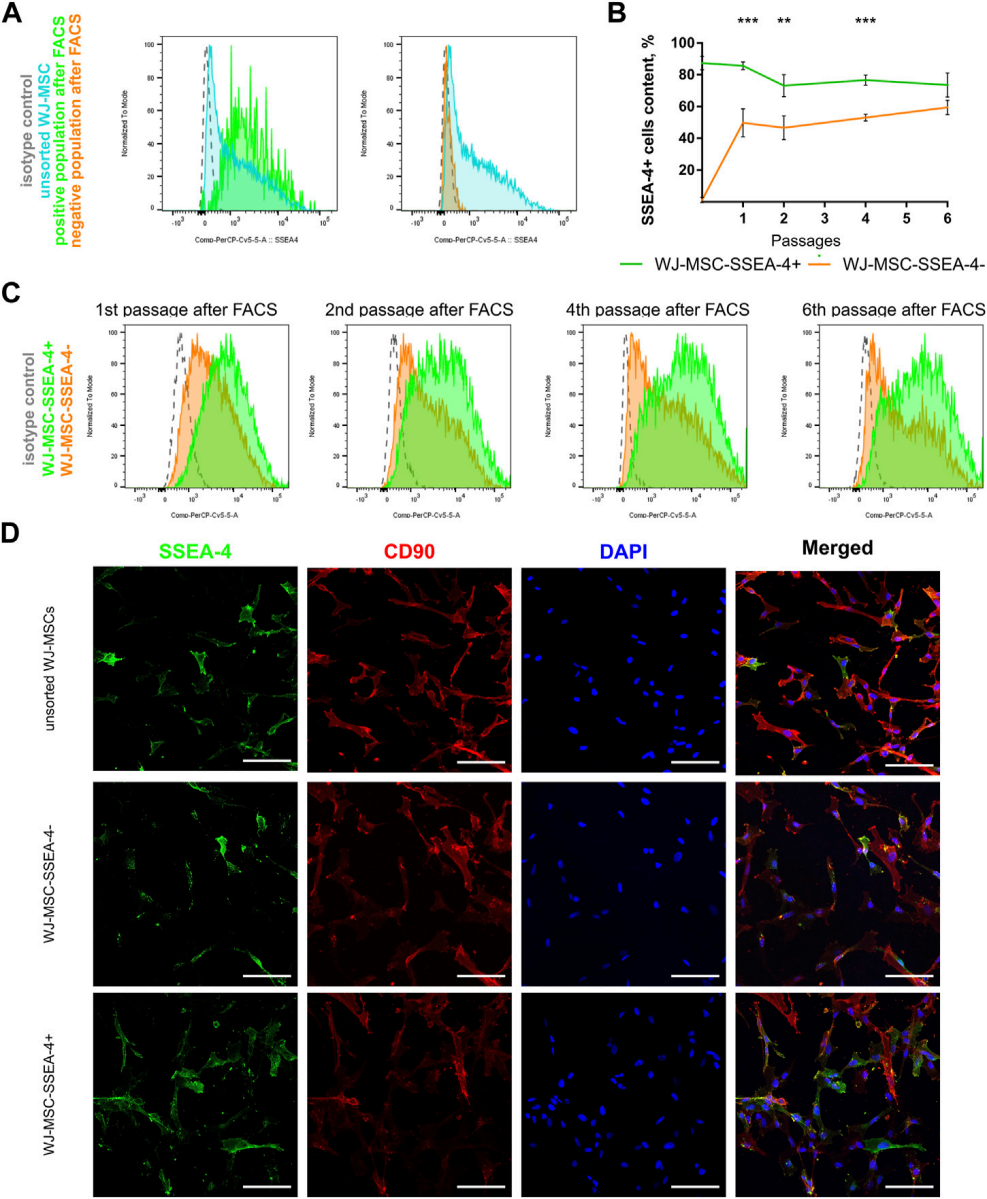
Persistence of SSEA-4+ cells after FACS in WJ-MSC populations: unsorted WJ-MSCs, WJ-MSC-SSEA-4+, and WJ-MSC-SSEA-4-. **(A)** Purity and yield of FACS—the comparison of unsorted WJ-MSCs before FACS and in positive and negative populations after FACS; representative histograms. **(B)** SSEA-4+ cells content dynamics during cell culture after FACS. The results are presented as mean values of three experiments ±SD, *p*-value for ** <0.01 and *** <0.001. **(C)** SSEA-4+ cell content changes after 1, 2, 4, and 6 passages after FACS; representative histograms. **(D)** SSEA-4 co-expression with CD90 for unsorted populations and positive and negative populations at the end of the first passage after FACS. White scale bars indicate 100 µm.

As a result of FACS, two groups of cells were received, which are the negative subpopulation (WJ-MSC-SSEA-4-) and the positive subpopulation (WJ-MSC-SSEA-4+) (Figure 3). The content in the unsorted population before FACS and in populations received after FACS was measured to examine purity and yield (Figure 3A). The FACS method resulted in 87.4% ± 4.3 of SSEA-4+ cells in the positive population, while the negative population contained 1.2% ± 1.6 of SSEA-4+ cells (Figure 3B). SSEA-4+ cell content in positive and negative populations was monitored for the next six passages (Figures 3B,C). For the first four passages, both populations differed significantly in SSEA-4+ percentage. SSEA-4+ cell content was observed to increase in the negative population. In the sixth passage after FACS, no differences between the analyzed populations were recorded. Immunocytochemical staining revealed notably more SSEA-4+ cells in the positive population after FACS separation (Figure 3D).

### 3.4 Pluripotent-like properties of WJ-MSC-SSEA-4+ subpopulation

**FIGURE 4:**
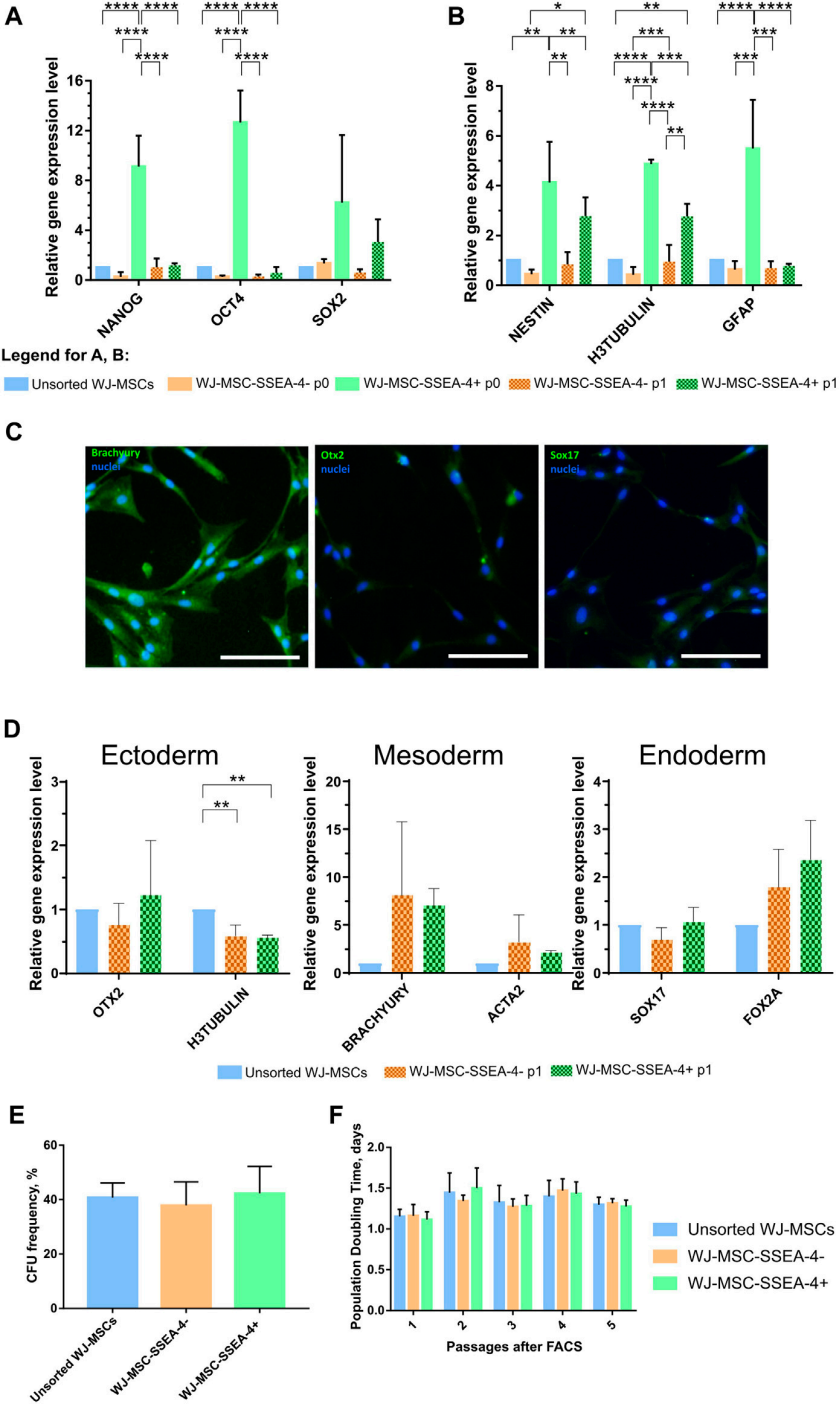
Stemness properties of WJ-MSC populations. **(A, B)** Relative gene expression level (fold change, mean ± SD) of pluripotency **(A)** and neural **(B)** associated genes. Quantitation was determined relative to ACTB by quantitative real-time PCR. Changes in gene expression are shown relative to the unsorted WJ-MSC populations (value = 1). The expression of the following groups was analyzed: unsorted WJ-MSCs, negative fraction collected directly after sorting (WJ-MSC-SSEA-4- p0), positive fraction collected directly after sorting (WJ-MSC-SSEA-4+ p0), negative fraction cultured for one passage *in vitro* (WJ-MSC-SSEA-4- p1), and positive fraction cultured for one passage *in vitro* (WJ-MSC-SSEA-4+ p1). The results shown are the mean of three independent RNA isolations, with the *p*-value of * <0.05, ** <0.01, ***<0.001, and ****<0.0001. **(C)** Immunocytochemical analysis of Brachyury, Otx2, and Sox17 (green) expression. Nuclei were counterstained with DAPI (blue). Scale bar = 100 μm. **(D)** The relative gene expression level (fold change, mean ± SD) of genes associated with specific germ layers, OTX2 (ectoderm), BRACHYURY (mesoderm), and SOX17 (endoderm), after differentiation. Quantitation was determined relative to ACTB by quantitative real-time PCR. Changes in gene expression are shown relative to the unsorted WJ-MSC populations (value = 1). The results shown are the mean of three independent RNA isolations. **(E)** Colony forming unit (CFU) frequency assay. **(F)** Population Doubling Time (PDT). The results are presented as mean values of three experiments ±SD.

The expression of genes associated with pluripotent stem cells (Nanog, Oct4, and Sox2) was compared to confirm the undifferentiated state of the SSEA-4+ population (Figure 4A). RNA was collected directly after cell sorting (p0) and after one passage of *in vitro* cell culture (p1) for the populations received with FACS. WJ-MSC-SSEA-4- + population exhibited increased expression of Nanog and Oct4 directly after cell sorting, which decreased with cell culture. The expression of early neural genes connected with the ectoderm germ layer was also analyzed (Figure 4B). An increased expression of H3TUBULIN, NESTIN, and GFAP was observed in the positive population directly after FACS sorting; the expression of H3TUBULIN was still elevated after one passage of cell culture.

Subsequently, the potential to differentiate toward cells derived from three germ layers was compared between unsorted and sorted populations (Figures 4C,D; Supplementary Figure S4). Final effects were evaluated with the measurement of specific gene expression–OTX2 for ectodermal differentiation, BRACHYURY for mesodermal differentiation, and SOX17 for endodermal differentiation–and visualized with immunocytochemical staining. Both populations received in FACS sorting, WJ-MSC-SSEA-4+, and WJ-MSC-SSEA-4- exhibited increased expression of BRACHYURY, compared to the unsorted population, but the differences were not statistically significant. Unchanged OTX2 and SOX17 expression indicated that the WJ-MSC-SSEA-4+ population lacked pluripotent potential (Figure 4D). The immunocytochemical analysis confirmed the abovementioned results: high expression for the mesodermal marker (Brachyury) and no clear expression for ectodermal and endodermal lineage. No difference between all three populations was observed (Figure 4C). Additionally, the observed mesodermal differentiation capacity toward osteocytes, adipocytes, and chondrocytes was similar for both analyzed populations (Supplementary Figure S10).

The physiological properties of the analyzed subpopulations–clonogenicity and proliferation–between passages were also investigated. Clonogenicity was examined with the CFU assay (Figure 4E). Proliferation was described as the PDT for five passages after FACS (Figure 4F). No significant differences were recorded between unsorted, negative, and positive subpopulations in the CFU assay and PDT measurements. With regard to proliferation and clonogenicity, the SSEA-4+ population propagated in the *in vitro* culture as was the case in the negative and initial populations.

### 3.5 Expression of other surface antigens within WJ-MSC populations after SSEA-4+ enrichment

**FIGURE 5:**
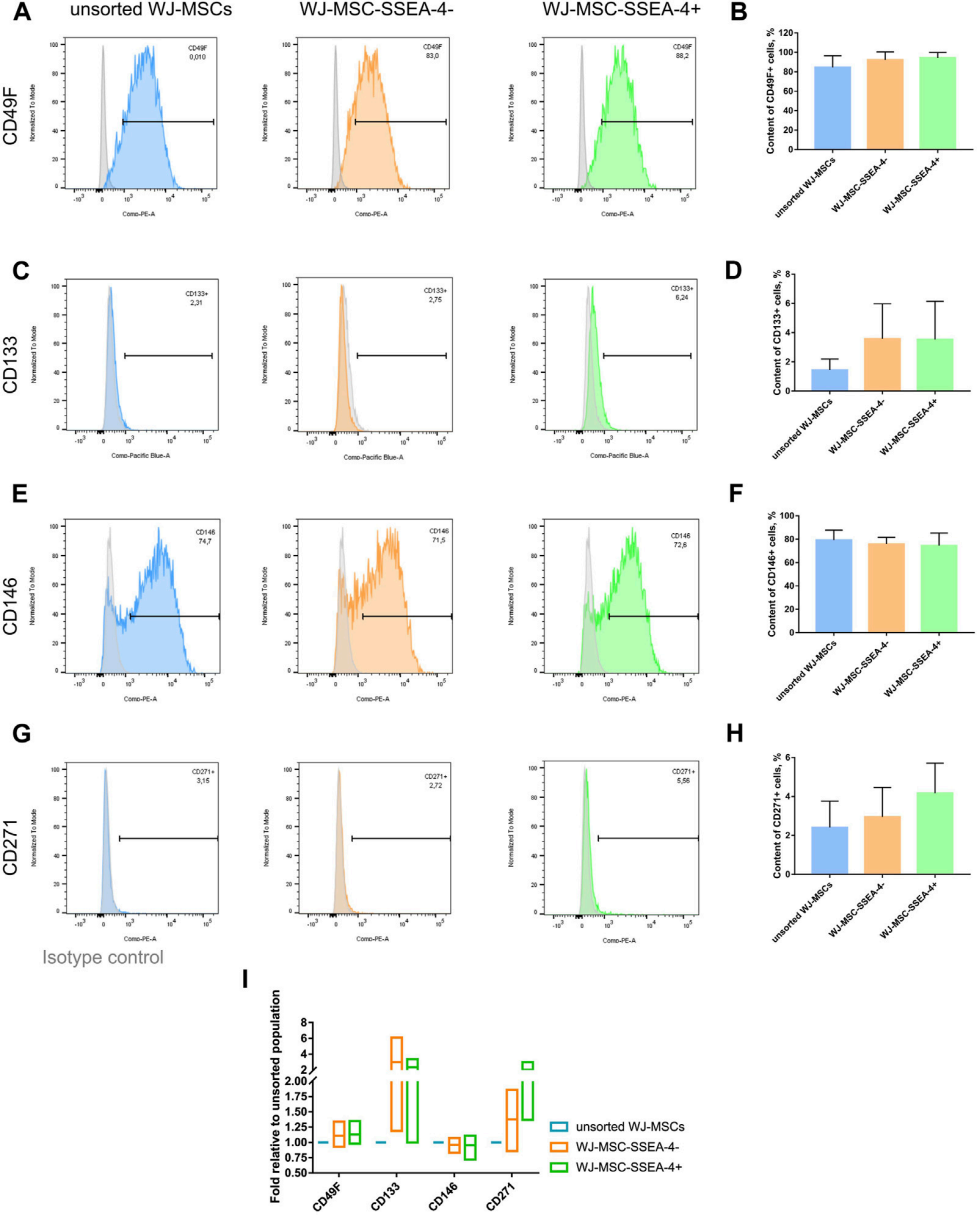
Unique surface antigens before and after FACS for the following populations: unsorted WJ-MSCs, WJ-MSC-SSEA-4- and WJ-MSC-SSEA-4+. **(A–H)**. Flow cytometry analyses – representative histograms and gathered results for CD49F **(A, B)**, CD133 **(C, D)**, CD146 **(E, F)**, CD271 **(G, H)**. Grey histogram – isotype control. **(I)**. Change in the content of the analyzed antigens after cell sorting compared to the content observed in the unsorted population. For **(B, D, F, H and I)** the results are presented as mean values of 3 experiments ± SD.

We examined whether the SSEA-4+ cell enrichment influenced the expression of other surface antigens associated with other stem cells still found within MSC populations, which are CD49F, CD133, CD146, and CD271 (Figure 5). We did not observe significant changes in the percentage of the surface antigens before and after cell sorting. WJ-MSC subpopulations contained 84%–94% of CD49F + cells (Figures 5A,B), 1.4%–3.5% of CD133+ cells (Figures 5C,D), 74%–79% of CD146+ cells (Figures 5E,F), and 2.4%-4% of CD271+ cells (Figures 5G,H). For each antigen, the fold in expression was calculated in relation to the unsorted population (Figure 5I). The most significant changes were observed for antigens that were sparse in population, such as CD133 and CD271. However, we did not record any statistically significant differences again, potentially due to considerable discrepancies between samples received from different donors. Additionally, the same analysis was performed for AD-MSCs isolated from adult donors and a significantly lower number of CD49F cells (34.2% ± 14.5) was found, while the CD271+ subpopulation was more numerous than in the WJ-MSCs (32.4% ± 7.8) (Supplementary Figure S11). Our surface antigen analysis revealed that the SSEA-4+ population could hardly be connected with other unique subpopulations.

### 3.6 Secretory profiles of subpopulations

**FIGURE 6:**
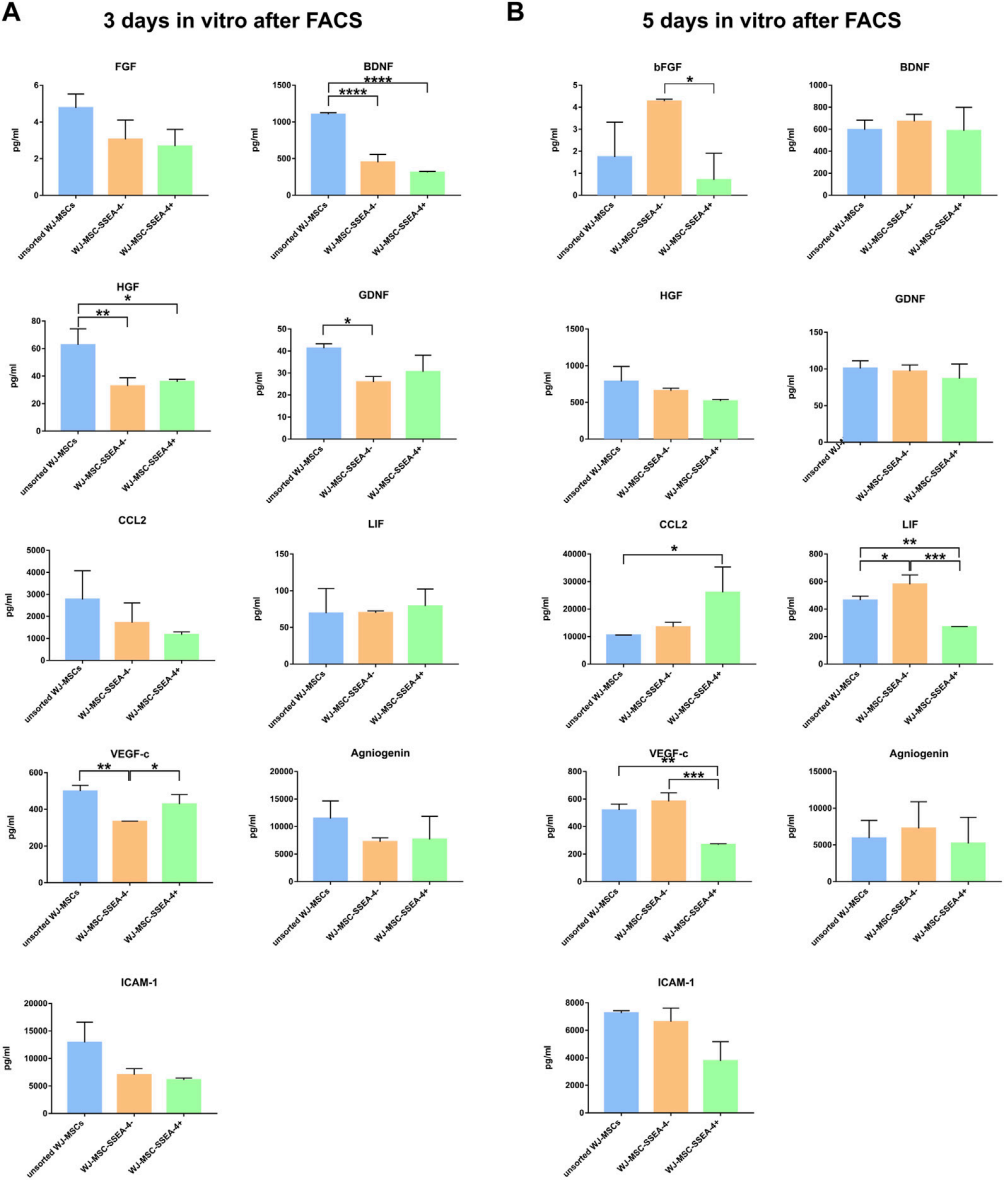
Secretory profiles 3 days *in vitro* **(A)** and 5 days *in vitro* **(B)** after FACS. The following groups were analyzed: unsorted WJ-MSCs, negative population (WJ-MSC-SSEA-4-), and positive population (WJ-MSC-SSEA-4+). The results are presented as mean values ±SD of three experiments. *p*-value for * <0.05, ** <0.01, *** <0.001, and **** <0.0001.

In the next step, we compared the secretory profiles of unsorted WJ-MSCs, WJ-MSC-SSEA-4-, and WJ-MSC-SSEA-4+ subpopulations on days 3 and 5 *in vitro* after FACS sorting (Figure 6). We measured the levels of selected trophic factors (EGF, bFGF, GDNF, and BDNF), cytokines and chemokines (CCL2 and LIF), and vascular factors (angiogenin, VEGF-c, and ICAM-1). The unsorted population exhibited an increased secretion of BDNF, HGF, and GDNF on the third day of our observation. The WJ-MSC-SSEA-4- population secreted less VEGF-c on the third day after FACS sorting. The secretion profile slightly differed on the fifth day after FACS sorting. The WJ-MSC-SSEA-4- population secreted higher levels of bFGF and LIF, compared to other variants. The WJ-MSC-SSEA-4+ population secreted a higher level of CCL2 and a lower level of VEGF-c. Except for soluble molecules described above, no significant differences in secretion of other factors were recorded between compared groups while the levels of EGF were found to be lower than the levels observed in the culture media of negative controls (Supplementary Figure S12).

### 3.7 Sphere formation ability in the WJ-MSC-SSEA-4+ population

**FIGURE 7:**
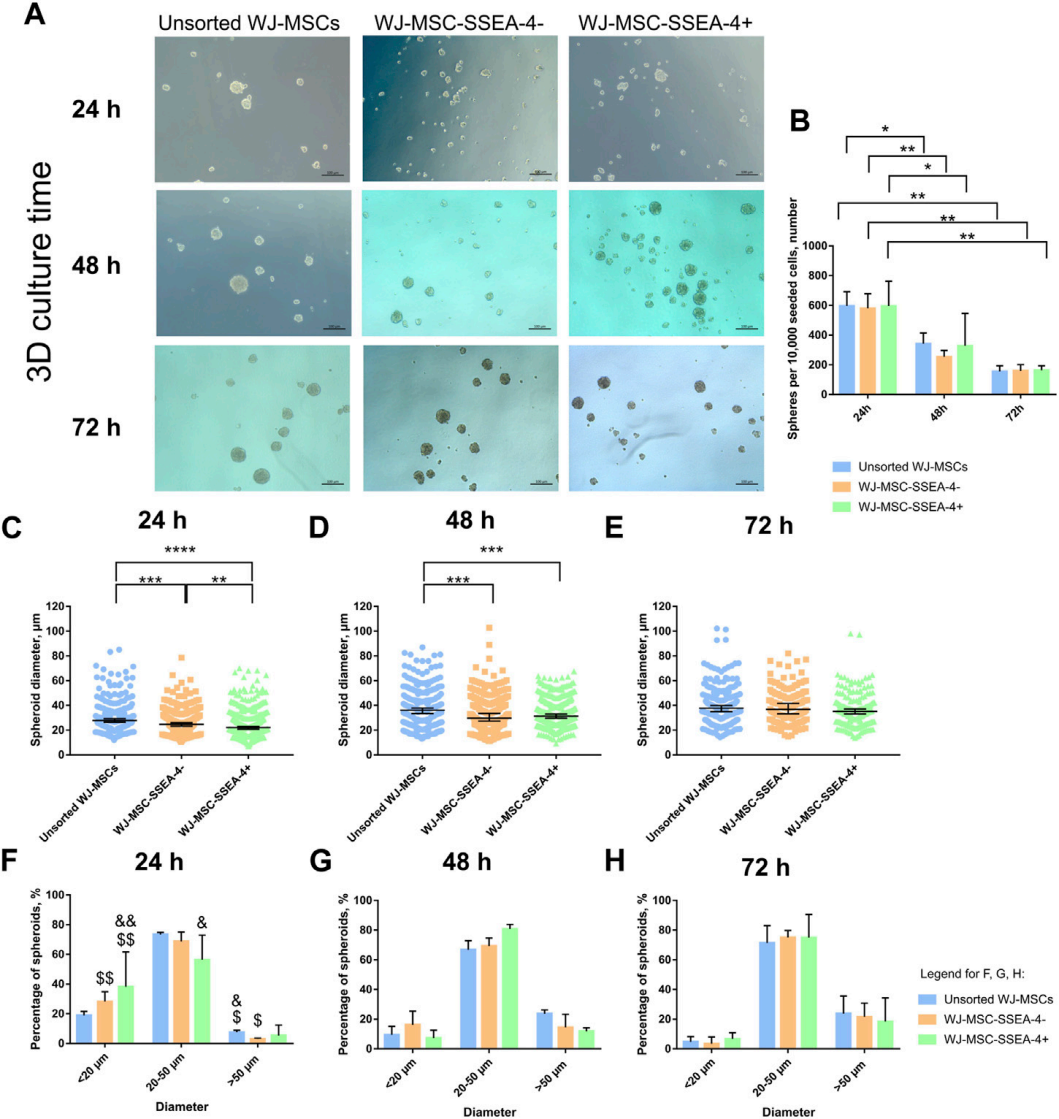
3D spheroid culture for unsorted WJ-MSCs, negative population (WJ-MSC-SSEA-4-), and positive population (WJ-MSC-SSEA-4+): time characteristics. **(A)** Morphology during the first 72 h of spheroid culture. Black scale bars represent 100 µm. **(B)** The number of spheroids formed from 10,000 cells during the first 72 h of spheroid culture. **(C–E)**: spheroid diameter during 24, 48, and 72 h of 3D culture, respectively. **(F–H)**: percentage of spheroids depending on the diameter during 24, 48, and 72 h of 3D culture, respectively. The results are presented as mean values ±SD **(B, F, G, and H)** or median values ±95% Confidence Interval **(C–E)** of three experiments. *p*-value for * <0.05, ** <0.01, *** <0.001, and **** <0.0001; significant differences for **(F–H)**: & - 24 h vs. 48 h, for $ - 24 h vs. 72 h, single symbol – *p*-value <0.05, double symbol – *p*-value <0.01.

Finally, we analyzed the influence of WJ-MSC-SSEA-4+ enrichment on the sphere formation ability in the 3D culture. The 3D culture was carried out with anti-adherent culture plates for 72 h *in vitro*. All the analyzed populations were observed to form spheroids with a small diameter (15–50 µm) (Figure 7A). For each day, we counted the cell number per 10,000 seeded cells (Figure 7B) and measured their diameters (Figures 7C–E); spheroids were grouped according to their size: small spheres (smaller than 20 µm), medium spheres (20–50 µm), and large spheres (larger than 50 µm) (Figures 7F–H). A decrease in spheroid number was observed in 3D culture (Figure 7B). After the first 24 h, WJ-MSC-SSEA-4- cells formed significantly smaller spheroids than the unsorted subpopulation (95% CI, 25.3–27.6 vs. 29.15–32.4, respectively), while WJ-MSC-SSEA-4+ cells were found to form the smallest spheroids (95% CI range: 23.4–25.6 µm) (Figure 7C). After 48 h of 3D culture, unsorted WJ-MSCs formed significantly bigger spheres than negative and positive populations (95% CI ranges, 35.89–39.47 vs. 31.41–34.82 vs. 31.65–34.03, respectively) (Figure 7D). After 72 h, no significant differences were detected in the diameter of the spheroid between the analyzed variants (Figure 7E). The medium-sized spheroid predominated in all the compared groups in 3D culture (Figures 7F,G). The percentage of spheres of different sizes varied over time. A significantly larger number of small spheroids was identified in negative and positive subpopulations during the first 24 h than in subsequent days of 3D culture. Similarly, we observed fewer medium-size spheres in the positive subpopulations and larger spheres in unsorted and negative subpopulations. WJ-MSC-SSEA-4+ cells formed smaller spheres during the first 24 h of 3D culture; however, the differences diminished with the duration of 3D culture.

**FIGURE 8:**
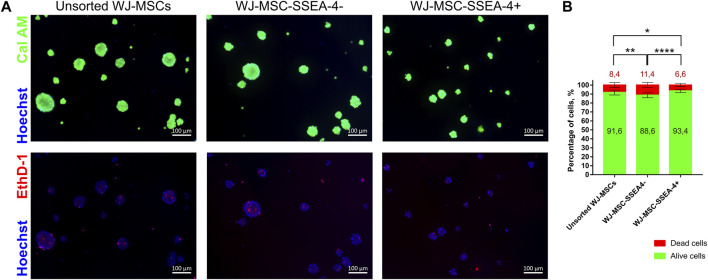
Viability of populations: unsorted WJ-MSCs, WJ-MSC-SSEA-4-, and WJ-MSC-SSEA-4+ after 72 h of 3D spheroid culture. **(A)** Calcein AM (Cal AM), ethidium homodimer-1 (EthD-1), and Hoechst staining. Green signals indicate alive cells (Cal AM), red signals indicate dead cells (EthD-1), and blue signals indicate nuclei (Hoechst). White scale bars represent 100 µm. **(B)** Analysis of dead and alive cell percentage in populations from CalAM-EthD-1 staining. The results are presented as mean values of three experiments ±SD. *p*-value for * <0.05, ** <0.01, and **** <0.0001.

The viability of cells after 3D culture was evaluated with live-dead staining using Cal AM, EthD-1, and Hoechst after 72 h of 3D culture (Figure 8A). Cal AM-stained live cells in green, while EthD-1 bonded with nucleic acid, indicating dead cells. Dead cells were mostly observed in the spheroid core. WJ-MSC-SSEA-4+ subpopulation contained significantly fewer dead cells than unsorted and negative subpopulations (6.6 ± 1.7 for WJ-MSC-SSEA-4+ vs. 8.4 ± 2.7 for unsorted WJ-MSCs vs. 11.4 ± 2.8 for WJ-MSC-SSEA-4-) (Figure 8B). Our experiments revealed that SSEA-4+ cells formed smaller spheres during the first 24 h of 3D culture; WJ-MSC-SSEA-4+ cells were characterized with better survival during 3D culture.

## 4 Discussion

The heterogeneity of the MSCs arises from multiple factors ranging from differences between donors, isolation sites, and methods to a variety of proposed culture conditions (Lech et al., 2016; Costa et al., 2021; Wedzinska et al., 2021). Moreover, the heterogeneity issue is even more multifaceted as the cells within an established *in vitro* culture differ in morphology, size, phenotype, and differentiation potency (Sun et al., 2020; Wang et al., 2021; Zhang et al., 2022). Confirmed differentiation of multipotent MSCs toward neuron-like cells could result from intrinsic cell plasticity or contamination by cells of different origins (Somoza et al., 2008). The heterogeneity of MSCs causes a serious limitation in the translation of MSC studies for further clinical research. The application of a homogeneous subpopulation of morphologically similar cells would not only overcome this problem but would also result in better therapeutic outcomes as separated cells could exhibit outstanding properties such as faster proliferation (Kawamura et al., 2018) or unique differentiation directions (Khaki et al., 2018). Separation by surface antigen is one of the ideas in search of such a promising subpopulation, especially because MSCs present a variety of markers associated with other cell types. This study investigated whether SSEA-4+ could distinguish genuine (pluripotent-like) stem cells within MSC populations, especially in light of our previous studies, in which this subpopulation survived significantly longer in 3D culture (in spheres) (Kaminska et al., 2021).

Heterogenous MSC populations can be divided at least into two distinct classes that differ significantly in terms of ontogenetic origin and relatedly basic biological characteristics. MSCs isolated from perinatal tissues, i.e., umbilical cord, umbilical cord blood, placenta, or amniotic fluid, are related to the early stages of fetal development and their spectrum of differentiation seems to be broader. The second class represents MSCs isolated from the adult tissue, e.g., bone marrow or adipose tissue, with lower clonogenic and proliferative potential (Drela et al., 2016). MSCs from neonatal and mid-gestational fetal tissues exhibit extremely low immunogenicity; they are more plastic and grow faster. WJ-MSCs also possess the spontaneous potential to express neural markers, which have almost been undetected in BMSC. The ontogenetic origin of “primitive” MSCs was described by Takashima et al. (Takashima et al., 2007), who used Cre recombinase-mediated lineage tracing analysis which revealed that a primitive class of immature somatic progenitors with pluripotent potential and a preference for neuronal differentiation may originate from the embryonic neural crest neuroepithelium. After undergoing the first developmental epithelial-mesenchymal transition (EMT), these cells could form a cohort of primitive mesenchymal cells (pre-MSCs) that transiently populate all fetal tissue niches and then are gradually replaced by the mesoderm-recruited, post-gastrulation, adult MSCs.

Based on the above data, in order to find the marker of “primitive MSCs”, we selected an ontogenetically younger mesenchymal stem/stromal cell source, i.e., umbilical cord stroma (Wharton’s jelly). WJ-MSCs used in our experiments expressed surface antigen characteristics of MSCs and possessed multipotent capacities to differentiate toward mesodermal cells (Supplementary Materials). On the basis of previous studies (Musiał-Wysocka et al., 2019), despite reports of slightly lower SSEA4 expression in 5% oxygen concentration, we decided to apply hypoxic/physioxic conditions that are considered closer to physiological conditions within the cell niche than atmospheric 21% concentration (Ivanovic, 2009). Following the flow cytometry analysis performed for MSCs derived from different tissues and passages, and cultured in different media, we decided to apply WJ-MSCs from the third passage cultured in platelet lysate with a higher concentration. The estimations of SSEA-4 expression in MSC populations vary across scientific literature and depend on several factors. Petrenko et al. reported differences between SSEA-4-cell percentage values observed in different MSC sources: 10% for AD-MSCs, 55% for bone marrow-derived MSCs (BM-MSCs), and 60% for WJ-MSCs (Petrenko et al., 2020). BM-MSCs from younger donors contained more SSEA-4+ cells (5.2% vs. 4% for elderly donors) (Kawamura et al., 2018) while MSCs isolated from female donors expressed fewer SSEA-4+ cells (72% vs. 79.8% observed in male donors) (Selle et al., 2022).

The available literature and our observations strongly suggest that the initial optimization should be vital for SSEA-4+ expression determination in MSC populations. Culture media components were also reported to affect SSEA-4 expression (He et al., 2014). He et al. confirmed that a higher concentration of fetal bovine serum increased SSEA-4+ cell content for WJ-MSC and BM-MSC (He et al., 2014), which is in agreement with our observation. It is especially important to emphasize this observation because sera and platelet lysates do differ between manufacturers and even batches. For the described experiments, we used PLT Gold for cell culture even though the analysis of its influence on MSC characteristics is lacking in scientific literature. However, studies performed for the older generations of this platelet lysate showed that it did not alter MSC characterization such as cell morphology, expression of MSC markers (CD73, CD90, and CD105), multipotent differentiation capacity, and proliferation ratio when compared with other human platelet lysates (Juhl et al., 2016; Lensch et al., 2018; Bhat et al., 2021). Low oxygen concentration is another factor that could reduce SSEA-4 expression (Musiał-Wysocka et al., 2019).In addition to environmental factors and the cell source, the technique of isolation may also be crucial. When isolating MSCs from WJ, the non-enzymatic method was reported to be the optimal one to obtain cells with higher clonogenic and proliferative potential expressing spontaneous neural markers (Lech et al., 2016). Unfortunately, this method applied in our experiments did not allow for an assessment of SSEA4 expression in freshly isolated, uncultured cells.

With our non-enzymatic method, it was not possible to analyze WJ-MSC without culture. To obtain the cells straight from the tissue, it would have been necessary to use the enzymatic method which does not seem to be optimal for UC-MSCs (Lech et al., 2016). In our so-called “0 passage” culture, the number of SSEA4+ cells varied and ranged from 30% to 90%. Other researchers reported that they isolated WJ-MSC from three patients and SSEA4+ cells accounted for 51%, 67%, and 70%, respectively (Musiał-Wysocka et al., 2019).

Furthermore, it is still debated whether MACS or FACS is a better sorting option to favorably affect the process efficiency and the cell quality. Some researchers reported that MACS allowed for the isolation of positive populations with reduced cell stress and increased yield (Bowles et al., 2019), while others found FACS-based selection less variable (Muratore et al., 2014; Cheng et al., 2017). FACS was shown to produce better outcomes in SSEA-4+ cell isolation from ESC populations (Fong et al., 2009). Sutermaster and Darling observed inefficient cell sorting and high false-negative rates when MACS was used according to the manufacturer’s protocols (antibody and microbead concentration). After optimization, however, comparable MACS and FACS outcomes were obtained (Sutermaster and Darling, 2019). A dual MACS-FACS sorting procedure was recorded as the most effective (Kerényi et al., 2016). Our study demonstrated that FACS resulted in a more satisfactory recovery. In our study, reduced mortality of cells and better yield after FACS sorting were recorded but the observed differences were not statistically significant. Ultimately, FACS processing was selected to separate SSEA-4+ cells as the method proved to result in better recovery of positive cells.

In this study, two populations were received with FACS separation: negative (WJ-MSC-SSEA-4-) and positive (WJ-MSC-SSEA-4+), and both were compared to unsorted WJ-MSCs. Post-sorting cytometric analysis to assess the purity was performed immediately after isolation. Increased values of SSEA-4+ percentage were recorded in the positive population and almost no SSEA-4+ cells were detected in the negative population directly after FACS. The purity of the following subpopulations was examined until the sixth passage of cell culture when we did not observe differences in the content of SSEA-4 + cells between the groups. Other research groups also reported the presence of SSEA-4+ cells in the negative population after cell sorting (Rosu-Myles et al., 2013; He et al., 2014). Although He et al. confirmed the purity of the negative population directly after cell sorting, they observed the SSEA-4 expression on similar levels both in positive and negative populations (He et al., 2014). Induction of SSEA-4 in a negative population could be associated with serum/platelet lysate concentration; this probably could provide an SSEA-4 substrate for cells. Rosu-Myles et al. reported a decrease in SSEA-4+ number during 28 days (approximately four passages) of culture in unsorted WJ-MSCs and both positive and negative subpopulations (Rosu-Myles et al., 2013). Glycosphingolipids, as well as other lipids, are not encoded by genes, while lipid cell composition is usually defined by enzymes involved in metabolic pathways (Dowhan, 2009). To detect SSEA-4 expression by qPCR, other studies used gene encoding sialyltransferase ST3GAL3 (ST3 beta-galactoside alpha-2,3-sialyltransferase 3) that is necessary for SSEA-4 synthesis (Hatzfeld et al., 2007). However, the main disadvantage of this approach is that it does not allow for direct measurement of SSEA-4 levels in cells.

Our quantitative PCR analysis revealed an increased expression of such stemness-related transcription factors as Oct4 and Nanog, which are associated with pluripotency. Small CD105+ SSEA-4+ cells isolated from bovine embryonic fibroblasts expressed pluripotent markers and differentiated toward cells from all three germ layers (Pan et al., 2015). He et al. did not observe an increased expression of pluripotency genes in the sorted SSEA-4+ subpopulation (He et al., 2014). In our earlier studies, we observed a spontaneous neural differentiation of MSCs manifested in the presence of genes and proteins associated with early neurons and glial cells (Figiel-Dabrowska et al., 2021; Tomecka et al., 2021). The SSEA-4+ population also exhibited increased expression of H-III-Tubulin, Nestin, and GFAP, thereby suggesting a better potential for ectoneural differentiation through their undifferentiated state. However, WJ-MSC-SSEA-4+ cells were incapable of efficient differentiation toward cells from all three germ layers, which definitely contradicts their pluripotent potential. Multipotential differentiation toward mesodermal lineage (osteocytes, adipocytes, and chondrocytes) was also performed by other researchers, but no notably significant differences were recorded in differentiation between cells from unsorted, positive, and negative populations (Rosu-Myles et al., 2013; He et al., 2014). He et al. did not only observe differences in proliferation between SSEA-4+ and SSEA-4- populations but also reported that SSEA-4+ expression was not correlated with cell proliferation (He et al., 2014). Furthermore, in our study, neither better proliferation nor colony-forming capacities by SSEA-4+ cells were detected, which is also consistent with other reports (He et al., 2014; Matsuoka et al., 2015). In contrast, Rosu-Myles et al. reported increased proliferation and clonogenicity potential for SSEA-4+ cells (Rosu-Myles et al., 2013). Interestingly, SSEA-4- subpopulation derived from limbal epithelial cells exhibited better clonogenicity than SSEA-4+ cells (Truong et al., 2011).

As MSCs are a highly heterogenous cell group, we investigated the impact of SSEA-4+ sorting on the expression of other surface antigens which have not been typically referred to by other researchers so far. We also assessed the expression of the markers in cells from two different sources: Wharton’s jelly and adipose tissue. Following the latest literature, the following markers were selected to be analyzed: CD49F, CD133, CD146, and CD271 (Bakondi et al., 2009; Krebsbach and Villa-Diaz, 2017; Wangler et al., 2019). The distribution of CD49F (integrin α6) in various stem cell populations suggests its involvement in the stemness (pluripotent) maintenance; it was identified on the surface of, i.a., embryonic stem cells, hair follicle stem cells, hematopoietic stem cells, neural stem cells, and some cancer stem cells (Krebsbach and Villa-Diaz, 2017). AD-MSC-CD49F + exhibited a greater proliferation and mesenchymal differentiation potential. In one of the available studies, mouse and rat AD-MSCs were found to contain a maximum of 30% CD49F + cells, depending on the culture of the passage (Zha et al., 2021). Contrastingly, in our study, almost 90% of the cells in WJ-MSC populations were CD49F while AD-MSCs contained only approximately 17% of CD49F + cells. To our knowledge, this is the first paper to report CD49F+ in the human MSC populations derived from neonatal sources. CD133 (prominin 1) is another surface antigen not only associated with cancer stem cells but also found on the surface of hematopoietic stem cells and neural stem cells (Glumac and LeBeau, 2018). CD133+ cells isolated from MSCs derived from peripheral blood and adipose tissue-derived MSCs were already reported to express pluripotent markers at a higher level than unsorted MSCs (González-Garza et al., 2018). In our study, CD133+ appeared sparse for both WJ-MSCs and AD-MSCs. The expression of CD146 (melanoma cell adhesion molecule - MCAM) is associated with vascular smooth muscle cell lineage commitment (Espagnolle et al., 2014). We observed a higher percentage of CD146+ cells in WJ-MSC populations than in AD-MSCs. Finally, we investigated the expression of CD271 (low affinity nerve growth factor receptor—LANGFR/p75), which indicates cells of neuroectodermal, neural crest origin (Sowa et al., 2013; Coste et al., 2017). CD271+ cells were self-renewed and differentiated into neurons and glial cells after transplantation *in vivo* (Morrison et al., 1999). MSC-CD271+ cells were found to exhibit faster proliferation and better clonogenicity and expression of pluripotent and neural genes (Mikami et al., 2011; González-Garza et al., 2018; Kawamura et al., 2018). Originally, we also intended to separate CD271 + subpopulation. However, the number of CD271+ within the WJ-MSC populations was not sufficiently high for all the planned analyses to be made. We found that SSEA-4+ enrichment enhanced the CD271+ cell population, but the differences between the groups were not statistically significant. SSEA-4+ sorting was not found to significantly affect the expression of the surface antigens, which could be explained by extensive deviations between cells isolated from different donors.

Secretory properties of MSCs have been widely investigated in the context of therapeutic application but a limited number of reports focused on the secretion abilities of specific MSC subpopulations. We tested the hypothesis that surface markers are strictly connected with stromal cell function by tuning the cytokines released (Islam et al., 2019) and modulating the tissue microenvironment. Here, we compared the correlation between the presence/lack of the SSEA4 marker and the levels of secreted, different regeneration-related molecules such as trophic factors, cytokines, chemokines, and factors associated with vasculogenesis. The secretion profiles were found to differ between the third and fifth day of the experiment. Reduced levels of some molecules (HGF, BDNF, and GDNF) observed in both sorted populations on the third day suggested the impact of FACS on the cells’ condition. On the fifth day after FACS, for some trophic factors, the highest levels of secretion were observed in the negative population and the lowest levels were recorded in the positive population, which suggests that the SSEA-4-deficient cells may be the population that is more specialized in the secretion of trophic factors. However, the large standard deviations imply that the factors secretion could be more of an individual matter, as reported in our other paper (Sypecka et al., 2022).

Our study also examined whether the WJ-MSC-SSEA-4+ population would exhibit better survival in 3D conditions. According to the latest literature, 3D conditions could resemble the native niche of MSCs more accurately than standardly used 2D culture systems and could be more effective in stemness maintenance (Jauković et al., 2020; Rybkowska et al., 2023). Our previous paper confirmed that long-term spheroid culture affected WJ-MSCs’ survival, proliferation, and senescence, as well as increased SSEA-4+ expression (Kaminska et al., 2021). In this study, we cultured WJ-MSCs from the analyzed groups for 3 days *in vitro* as spheroids and compared the number, diameter, and cell viability of the spheroids. Changes in diameter were recorded between variants for the first 48 h of 3D culture. The spheres formed with WJ-MSC-SSEA-4+ cells were the smallest in the first 24 h. At the endpoint, the differences between the groups ceased to be noticeable. SSEA-4+ cells derived from different tissues were also reported to form spheres (Barraud et al., 2007; Lopez-Lozano et al., 2022). SSEA-4+ isolated from the bovine embryonic fibroblast population formed larger spheres after the seventh day of 3D culture than SSEA-4 cells (Pan et al., 2015). The viability assay revealed that our WJ-MSC-SSEA-4+ subpopulation exhibited the lowest number of dead cells after 3D culture.

It would be an interesting aspect of the project if the differentiation potential of all analyzed populations cultured in 3D conditions could be assessed. Unfortunately, long-term 3D spheroids culture resulted in increased senescence and led to sphere disintegration of heterogenous WJ-MSCs (Kaminska et al., 2021).

Finally, the association of SSEA-4 with pluripotency was the last aspect we addressed in this study. Derivation of iPSCs from SSEA-3/4 knockout mice raised the question of whether SSEA-4 was essential (Hamamura et al., 2020). Moreover, the increased transient expression of pluripotent genes by the WJ-MSC-SSEA-4+ subpopulation did not affect the proliferation and colony-forming capacity. In fact, two states of pluripotency can be distinguished: naïve (observed for embryonic cells before implantation into the uterus) and primed (observed for cells after implantation) (Weinberger et al., 2016; Nishihara, 2017). SSEA-3 and SSEA-4 were associated only with primed pluripotency while SSEA-1 was observed in the naïve state (Nishihara, 2017). In both states, the cells were observed to express Nanog, Sox2, and Oct4 genes, and they did differentiate toward cells from all three germ layers and form teratomas *in vivo* (Nishihara, 2017). Knockout of B3GALT5, an enzyme involved in SSEA-3/4 synthesis, was reported to facilitate the transition of human ESCs from primed to naïve state (Lin et al., 2020). Contrastingly, SSEA-3+ cells isolated from the amniotic membrane appeared to represent a naïve state of pluripotency, which was confirmed by the presence of SSEA-1 and expression of KLF4—a gene characteristic only of this state (Ogawa et al., 2022). However, those observations were not confirmed in the SSEA-3+ population derived from other tissue sources. It remains debatable whether the presence of two states explains the observed results in the WJ-MSC-SSEA-4+ population.

Some limitations of this study should be acknowledged. A decrease in the cell viability observed in both methods of cell sorting is a downside of the methodology used. Slightly higher viability of cells from unsorted populations could influence the outcomes received in PDT and CFU assays. To minimize this effect, unsorted cells were transported to the sorting facility in similar conditions applied for both positive and negative subpopulations. Furthermore, if indeed the sorting procedure had such a profound effect on the condition of the cells, differences between passages in cell culture would have been noticed. The next limitation concerns the similarity of SSEA-4+ cell content between unsorted and positive populations. Most researchers report the results observed in positive and negative populations. We decided to analyze the outcomes from the initial population to confirm whether SSEA-4 enrichment indeed impacted WJ-MSC populations. Nevertheless, if SSEA-4 surface antigen had such a huge impact, we would have observed significantly different results at least in the negative population. In fact, some of the analyzed aspects such as proliferation, CFU, and expression of other surface antigens were almost similar in all study groups.

## 5 Conclusion

This study described the characteristics of SSEA-4+ cells separated from the heterogenous WJ-MSC populations. WJ-MSCs contained approximately 35%–70% SSEA-4+ cells, depending on the applied culture condition. The environment richer in proteins and trophic factors appeared to be more favorable for SSEA-4 cell enrichment probably due to providing the essential substrate for synthesis. FACS allowed for the selection of positive SSEA-4+ cells and its number increased during the further *in vitro* culture. Elevated relative expression of the investigated stemness-related genes suggested an undifferentiated state of the WJ-MSC-SSEA-4 + subpopulation, which could also affect the differentiation potential toward ectoneural cells. However, this effect was transient and diminished with further cell culture, which could account for the unchanged pluripotent differentiation potential, proliferation ratio, and colony-forming capacities observed in the positive population. The SSEA-4+ population was not found to be associated with other potential stemness surface antigens. SSEA-4 enrichment influenced such aspects of 3D culture as diameter during the first 24 h and viability of cells inside the spheres. Our hypothesis that WJ-MSC-SSEA-4+ cells could be a more favorable subpopulation due to unique pluripotent-like features and restorative/replacing properties could not be confirmed as no unequivocal results were obtained. However, the search for such a marker is an important direction for further research on mesenchymal stem cells.

## Data Availability

The datasets presented in this article are not readily available because of privacy issues to make sure that confidentiality of tissue’s donor is preserved. Requests to access the datasets should be directed to Anna Sarnowska (contact: asarnowska@imdik.pan.pl)

## Abbreviations

AD-MSCs, adipose-derived mesenchymal stem/stromal cells; BDNF, brain-derived neurotrophic factor; bFGF, basic fibroblast growth factor; Cal-AM, Calcein AM; CCL2, chemokine ligand 2; CFU, colony forming unit; EGF, epithelial growth factor; ESC, embryonic stem cells; EthD-1, Ethidium homodimer-1; FACS, Fluorescence Activated Cell Sorting (FACS); GDNF, glial cell line-derived neurotrophic factor; ICAM-1, intercellular adhesion molecule 1; iPSCs, induced pluripotent stem cells; LIF, leukemia inhibitory factor; MACS, Magnetic Activated Cell Sorting (MACS); MSCs, mesenchymal stem/stromal cells; PDT, population duplication time; RT-qPCR, Real Time-Quantitative Polymerase Chain Reaction; SSEA-4, Specific stage embryonic antigen-4; UC-MSCs, umbilical cord-derived mesenchymal stem/stromal cells; VEGF-c, vascular endothelial growth factor-c; WJ-MSCs, Wharton jelly mesenchymal stem/stromal cells; WJ-MSC-SSEA-4 +, SSEA-4-positive WJ-MSC population; WJ-MSC-SSEA-4-, SSEA-4-negative WJ-MSC population.
