# Supplementary figures and images for: Stemness properties of SSEA-4+ subpopulation isolated from heterogenous Wharton’s jelly mesenchymal stem/stromal cells
Source: Front Cell Dev Biol. 2024 Feb 22;12:1227034. doi: 10.3389/fcell.2024.1227034 (PMC10917976; doi:10.3389/fcell.2024.1227034)

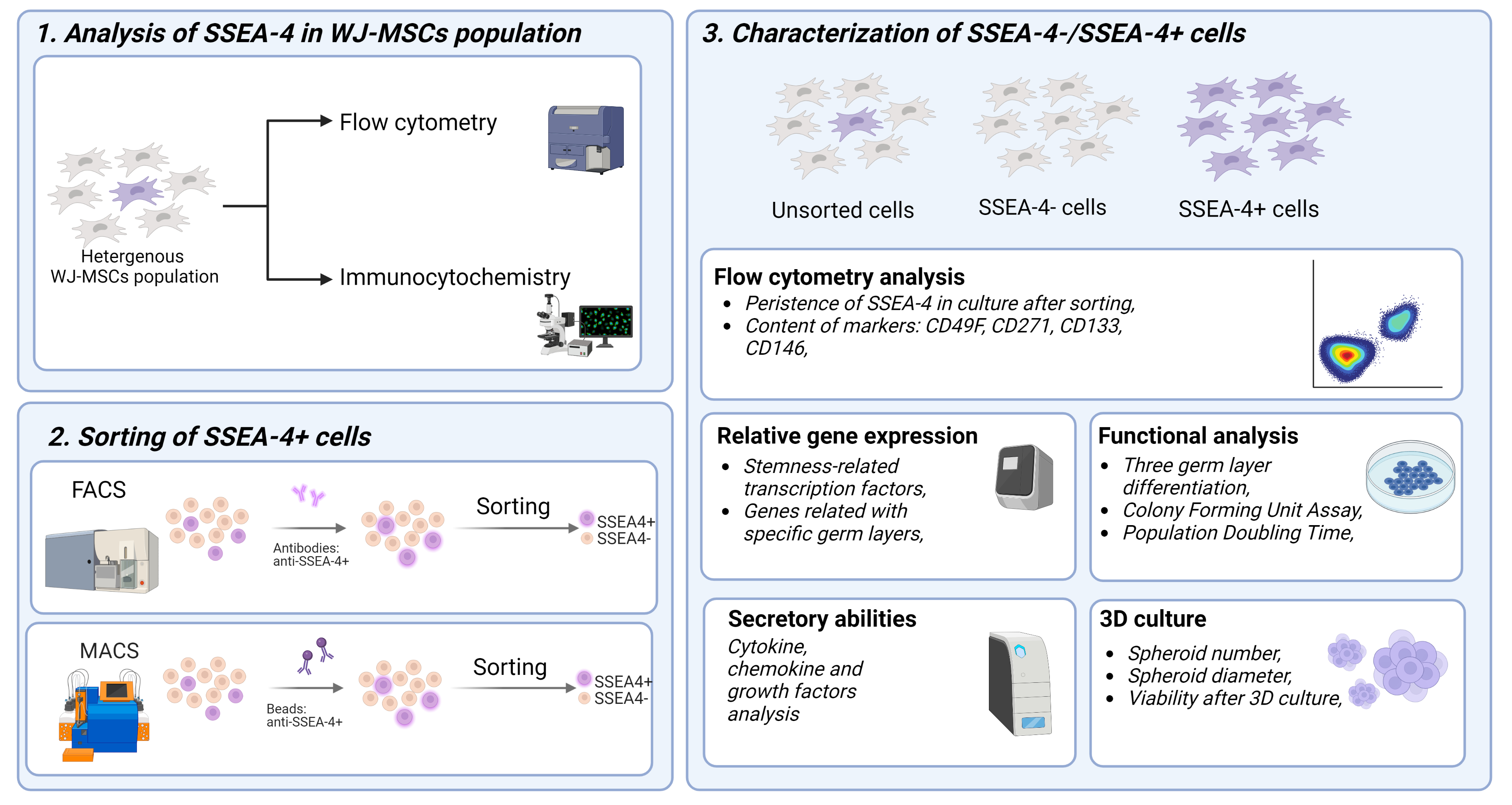

Supplement: Supplementary file 1 [file Image1.JPEG]
