# Supplementary material for: Stemness properties of SSEA-4+ subpopulation isolated from heterogenous Wharton’s jelly mesenchymal stem/stromal cells
Source: Front Cell Dev Biol. 2024 Feb 22;12:1227034. doi: 10.3389/fcell.2024.1227034 (PMC10917976; doi:10.3389/fcell.2024.1227034)
Supplement: Supplementary file 2 [file DataSheet1.docx]

Supplementary Material

Stemness properties of SSEA-4+ subpopulation isolated from heterogenous Wharton jelly mesenchymal stem/stromal cells First Agnieszka Smolinska, Magdalena Chodkowska, Agata Kominek, Jakub Janiec, Katarzyna Piwocka, Dorota Sulejczak, Anna Sarnowska*.

*** Correspondence:** Corresponding Author: [asarnowska@imdik.pan.pl](mailto:asarnowska@imdik.pan.pl)

# Supplementary materials and methods

**Materials and methods S1.** Flow cytometry analysis of MSCs specific surface antigens. For estimation of surface antigens content recommended by The International Society for Cellular Therapy, we used Human MSC Analysis Kit (BD) containing following anti-human antibodies: CD73-APC, CD90-FITC, CD105-PerCP-Cy5.5 (positive cocktail), CD11b-PE, CD19-PE, CD34-PE, CD45-PE (negative cocktail). According to the manufacturer protocols, cells were detached with Accutase Cell Detachment Solution (BD) and washed in PBS. Required cell number (1*106) was resuspend in cold Stain Buffer (BD) and then incubated with antibodies in the dark for 30 minutes. After incubation, cells were washed twice with Stain Buffer (BD) and resuspend in Stain Buffer. Resuspended cells were analysed using FACS Canto II (BD) with FACSDiva Software (BD) and FlowJo 10 (BD).

**Materials and methods S2.** MSCs multipotent differentiation assay. To verify the multipotency of WJ-MSCs used for experiments, mesodermal lineage differentiation was tested. Osteogenic, chondrogenic and adipogenic differentiation was induced with commercial differentiation media (Gibco, Thermo Fischer Scientific). Induction of adipogenesis and chondrogenesis was conducted for 14 days and induction for osteogenesis was conducted for 21 days. Then, cells were fixed in 4% PFA and then stained with histochemical dyes; osteogenesis was evaluated with 2% alizarin red S, chondrogenesis was evaluated with 1% alcian blue, adipogenesis was evaluated with 0,5% Oil Red.

**Supplementary Table S1.** List of primers used for RT-qPCR

| **Gene** | **NCBI Reference Sequence** | **Product size** | **Primer sequence (5’ -> 3’)** |
| --- | --- | --- | --- |
| β-Actin | NM_001101.5 | 250 bp | F: CATGTACGTTGCTATCCAGGC  R: CTCCTTAATGTCACGCACGAT |
| Nanog | NM_024865.4 | 103 bp | F: GAACCTCAGCTACAAACAGG  R: CGTCACACCATTGCTATTCT |
| Oct3/4 (Pou5F1) | NM_001285986.2 | 331 bp | F: CTGAAGCAGAAGAGGATCACC  R: AAAGCGGCAGATGGTCGTTTGG |
| Sox2 | NM_003106.4 | 93 bp | F: GTGGAAACTTTTGTCGGAGA  R: TTATAATCCGGGTGCTCCTT |
| Nestin1 | NM_006617.2 | 64 bp | F: GGGAAGAGGTGATGGAACCA  R: AAGCCCTGAACCCTCTTTGC |
| β-Tubulin III | NM_001197181.2 | 126 bp | F: GGAAGAGGGCGAGATGTACG  R: GGGTTTAGACACTGCTGGCT |
| GFAP | NM_001363846.2 | 100 bp | F: CCGACAGCAGGTCCATGT  R: GTTGCTGGACGCCATTG |
| OTX2 | NM_001270523.2 | 98 bp | F: TTCATGCGAGAGGAGGTGGCA R: TGCTGTTGTTGGCGGCACTT |
| Brachyuri | NM_001379200.1 | 104 bp | F:ACGGCCACATTATTCTGAAT R:GAAGTTCTCCTCGGCATATT |
| ACTA2 | NM_001141945.2 | 99 bp | F: CATCATGCGTCTGGATCTG  R: TCACGCTCAGCAGTAGTA |
| SOX17 | NM_022454.4 | 110 bp | F:AACTATCCTGACGTGTGACA R:CAAAAACCCAGGAGTCTGAG |
| FOX2A | NM_021784.5 | 89 bp | F: GGGAGCGGTGAAGATGGA  R: TCATGTTGCTCACGGAGGAGTA |

# Supplementary figures


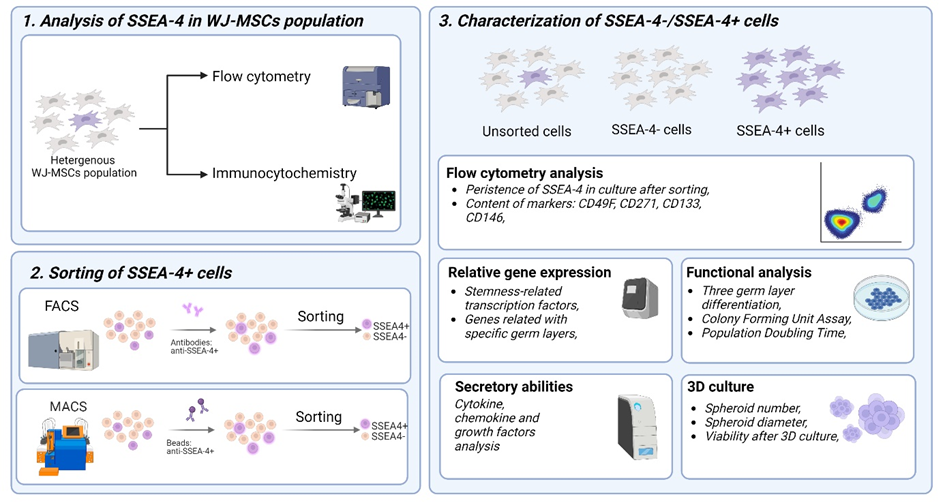


**Supplementary Figure 1.** General overview of experimental steps. First, SSEA-4 expression was analyzed within WJ-MSCs population. Then, two separation methods, Fluorescence Activated Cell Sorting (FACS) and Magnetic Activated Cell Sorting (MACS), were compared. Finally, SSEA-4+ population was characterized in comparison to the initial unsorted population and the negative population. The figure was created in BioRender (assessed date: 10.07.2023).


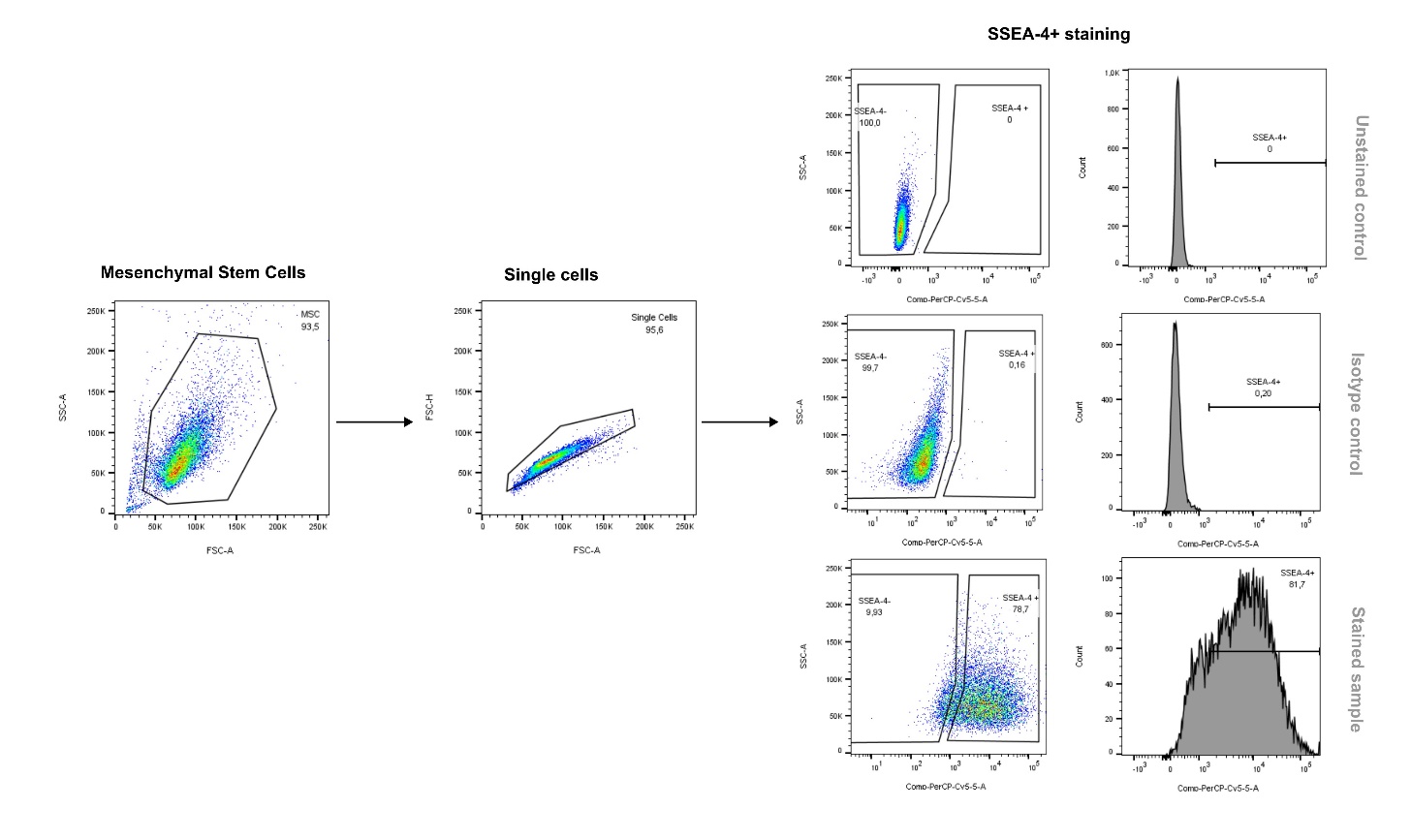


**Supplementary Figure 2**. Strategy gating for detection of SSEA-4+ cells in WJ-MSCs for unstained control, isotype control and stained sample; flow cytometry, FACSCanto II.


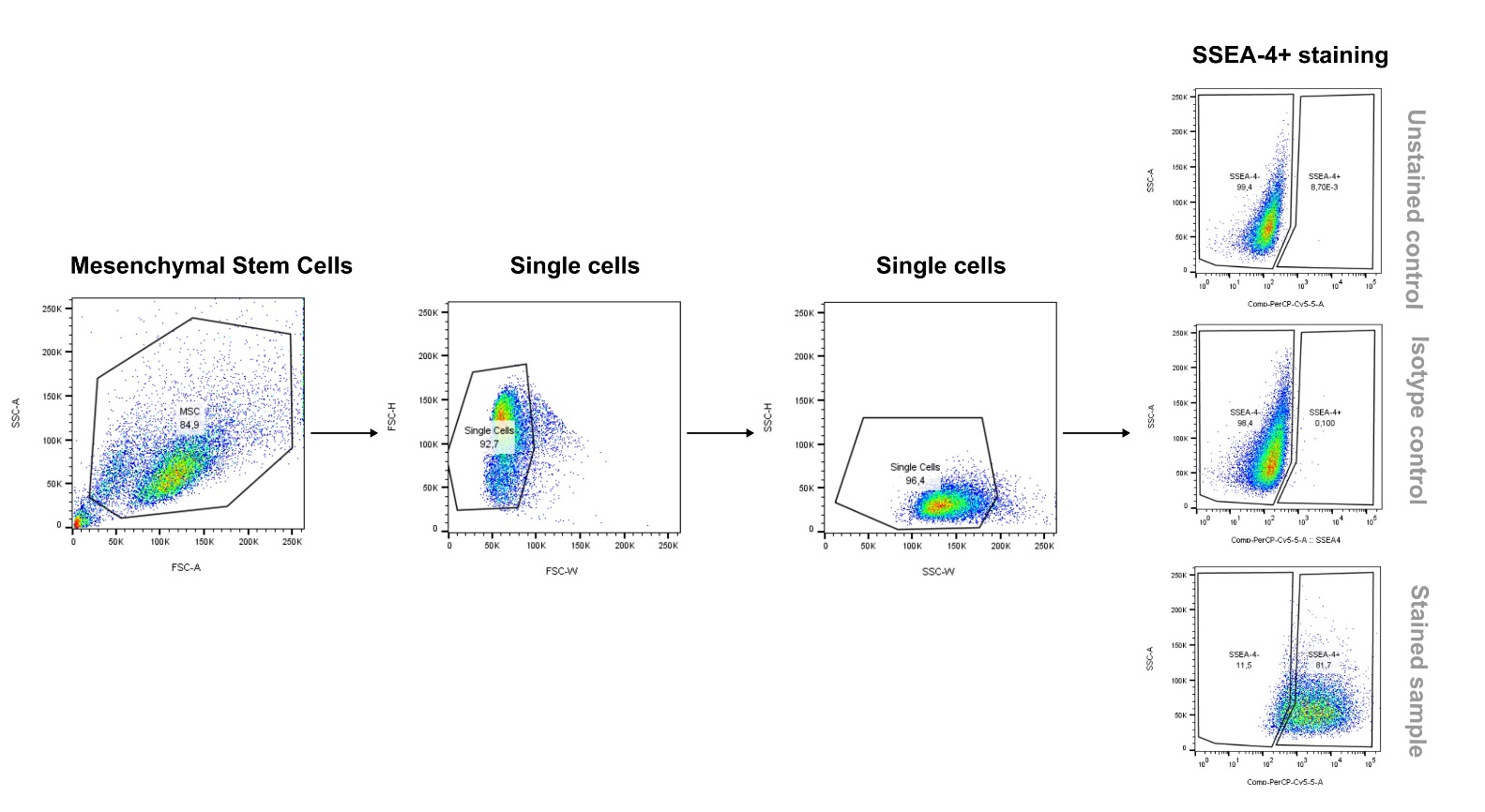


**Supplementary Figure 3**. Strategy gating for sorting of SSEA-4+ cells in WJ-MSCs for unstained control, isotype control and stained sample; flow cytometry, FACSAria II.

**
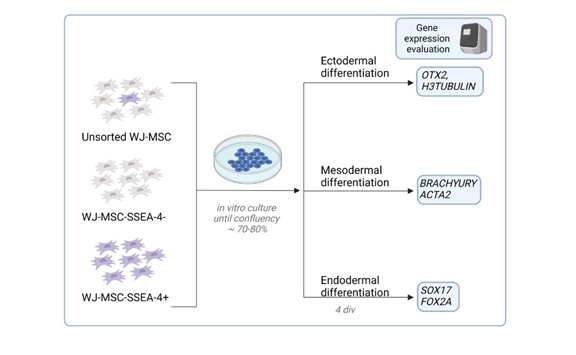
**

**Supplementary Figure 4**. Scheme of 3 germ layer differentiation. Cells from different variants were cultured until 70-80% confluency and then, culture medium was replaced with specific differentiation media. Then, the cells were cultured for 4 days in vitro (div) and then RNA was collected for gene expression analysis.


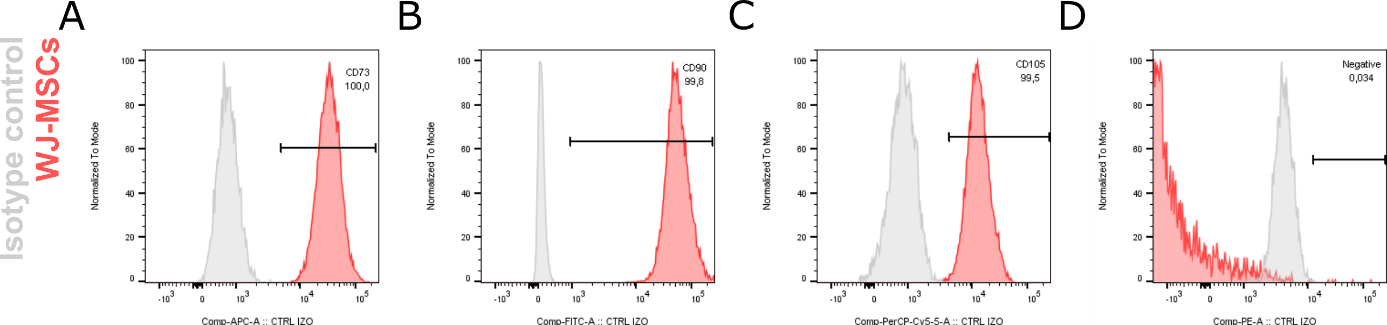


**Supplementary Figure 5**. Flow cytometry analysis of surface antigens recommended by *The International Society for Cellular Therapy* for MSCs characteristics – CD73 (A), CD90 (B), CD105 (C) and negative mix (CD11b, CD19, CD34, CD45 and HLA-DR).

| **Marker** | **Positive cells, % Mean** | **Standard deviation** |
| --- | --- | --- |
| CD73 | 99.7 | 0.40 |
| CD90 | 99.8 | 0.08 |
| CD105 | 97.4 | 1.90 |
| CD11b, CD19, CD34, CD45,  HLA-DR | 0.2 | 0.26 |

**Supplementary Table 2. Expression of surface antigens recommended by *The International Society for Cellular Therapy* for MSCs characteristics**


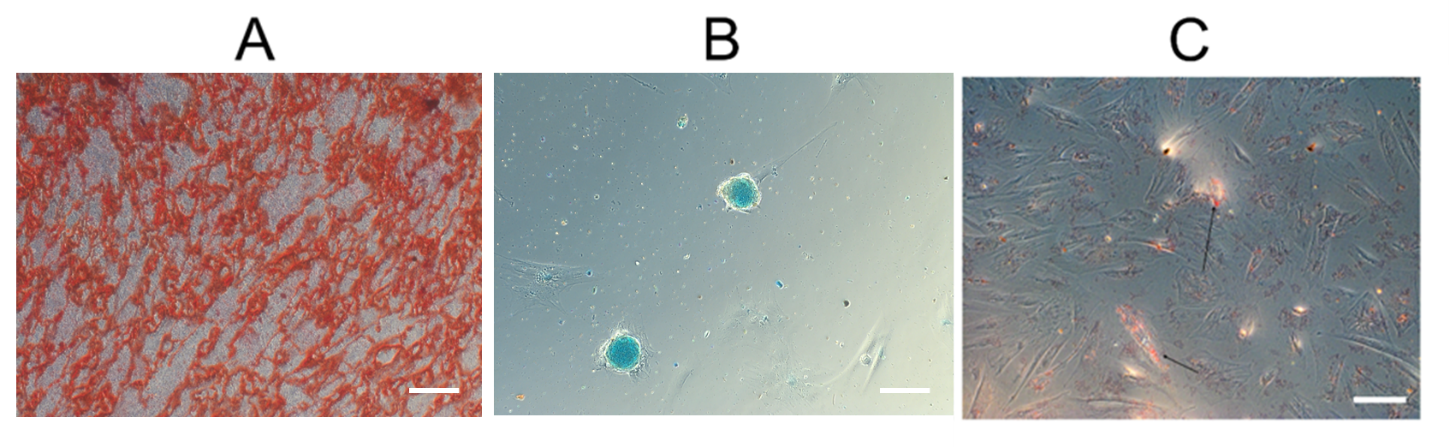


**Supplementary Figure 6**. Multipotent differentiation of WJ-MSC toward osteocytes (A), chondrocytes (B) and adipocytes (C). A. Calcium deposits characteristic for osteocytes were identified with alizarin red. B. Glycosaminoglycans characteristic for chondrocytes were identified with alcian blue. C. Lipid drops (black arrows) characteristic for adipocytes were identified with red oil. Scale bars: 100 µm.


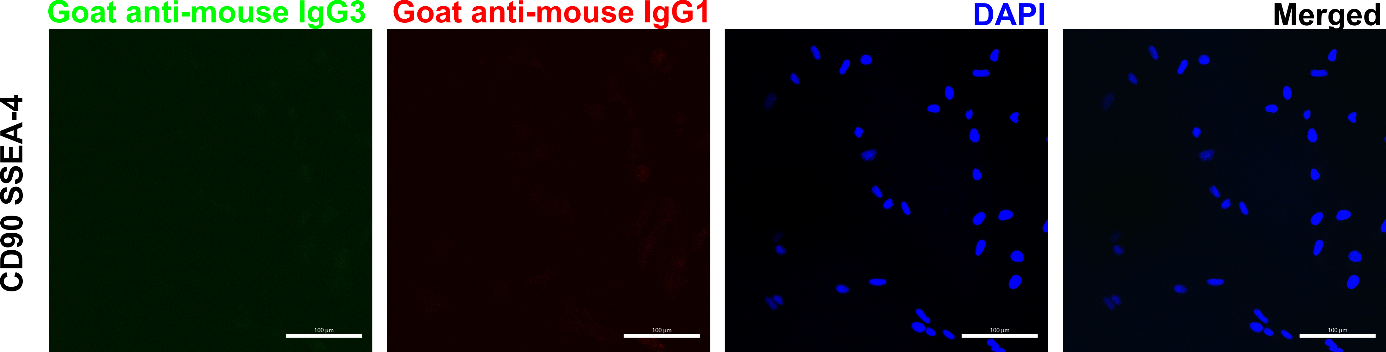


**Supplementary Figure 7**. Secondary antibody staining controls for Fig. 2.A. Following secondary antibodies were applied: goat anti-mouse IgG3 conjugated with Alexa Fluor 488 for SSEA-4, goat anti-mouse IgG1 conjugated with 546 for CD90. Cell nuclei were stained with DAPI. Scale bars: 100 µm.


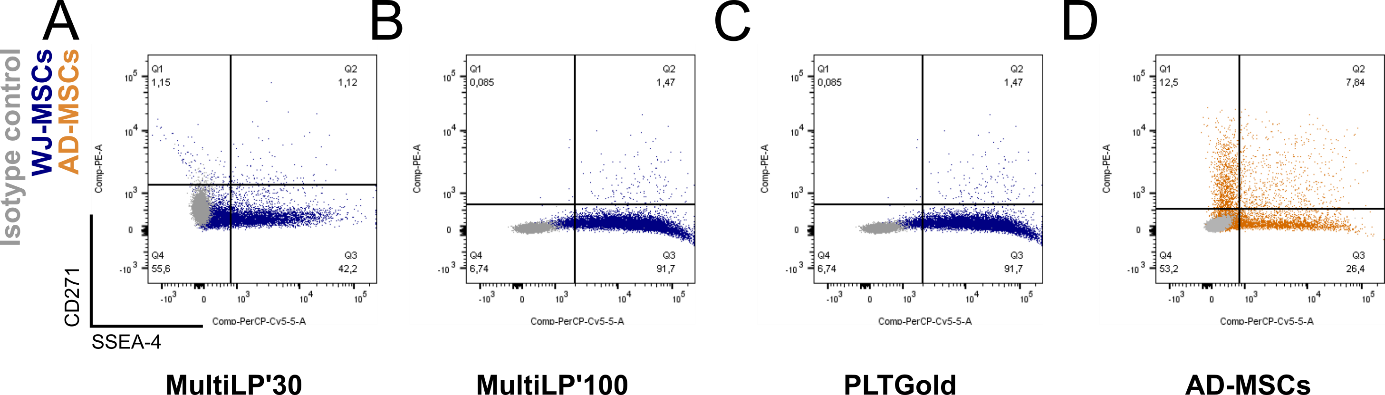


**Supplementary Figure 8**. SSEA-4 and CD271 coexpression, flow cytometry analysis. WJ-MSCs were cultured with following platelet lysates: MultiLP’30 (A) , MultiLP’100 (B) and PLTGold (C). AD-MSCs were cultured in PLTGold platelet lysate.


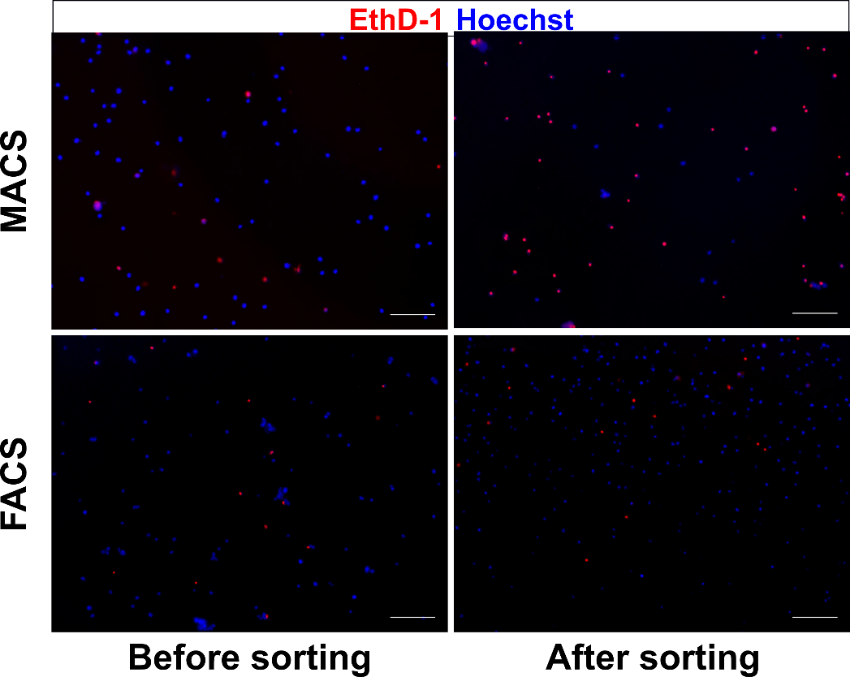


**Supplementary Figure 9**. Ethidium homodimer-1 (EthD-1) mortality staining for cells before and after cell sorting with MACS and FACS. Cell nuclei were stained with DAPI. Scale bars: 100 µm.


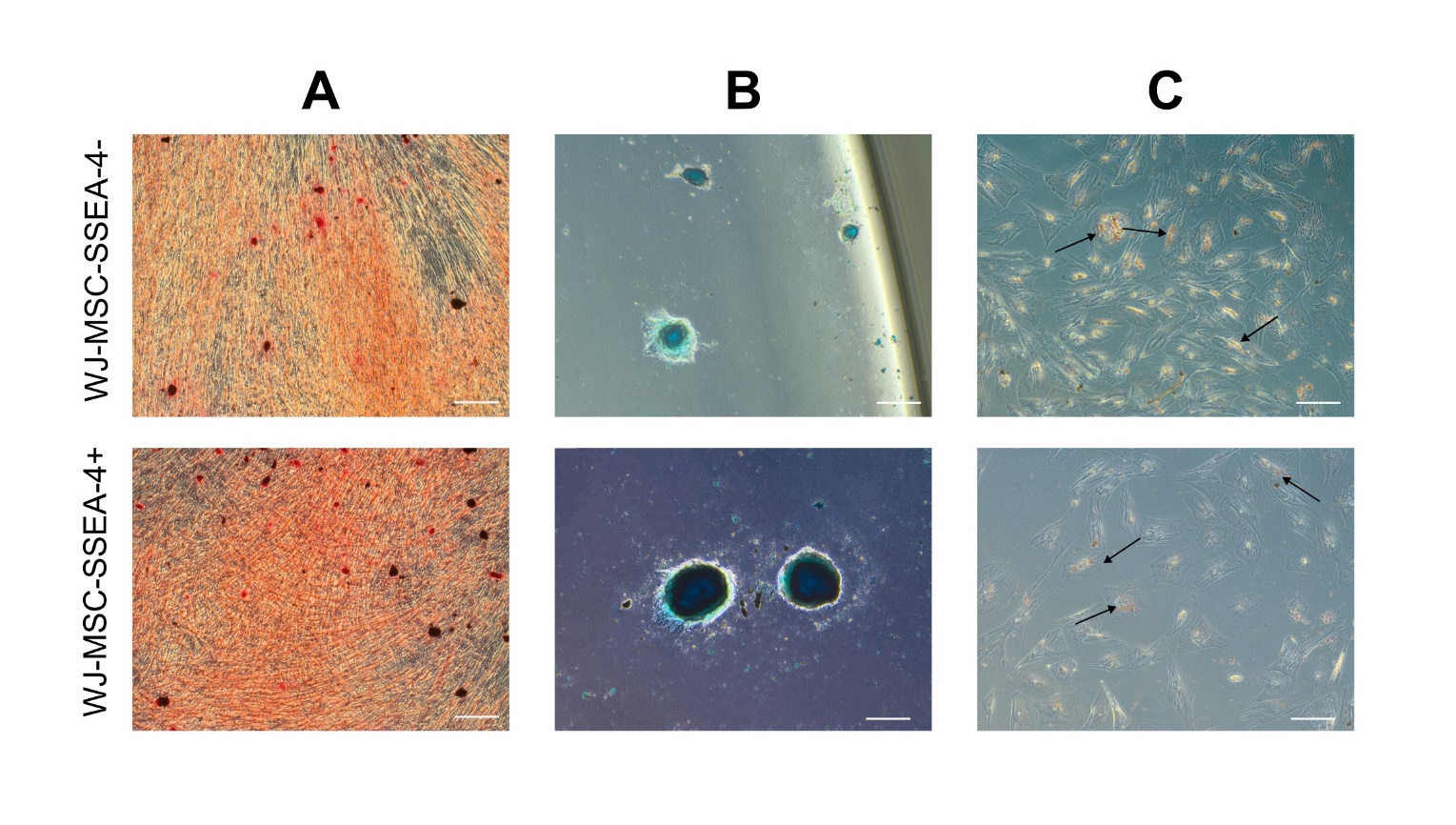


**Supplementary Figure 10**. Multipotent differentiation of SSEA-4 negative (WJ-MSC-SSEA-4-) and SSEA-4 positive WJ-MSC (WJ-MSC-SSEA-4+) populations toward osteocytes (A), chondrocytes (B) and adipocytes (C). Scale bars: 100 µm.


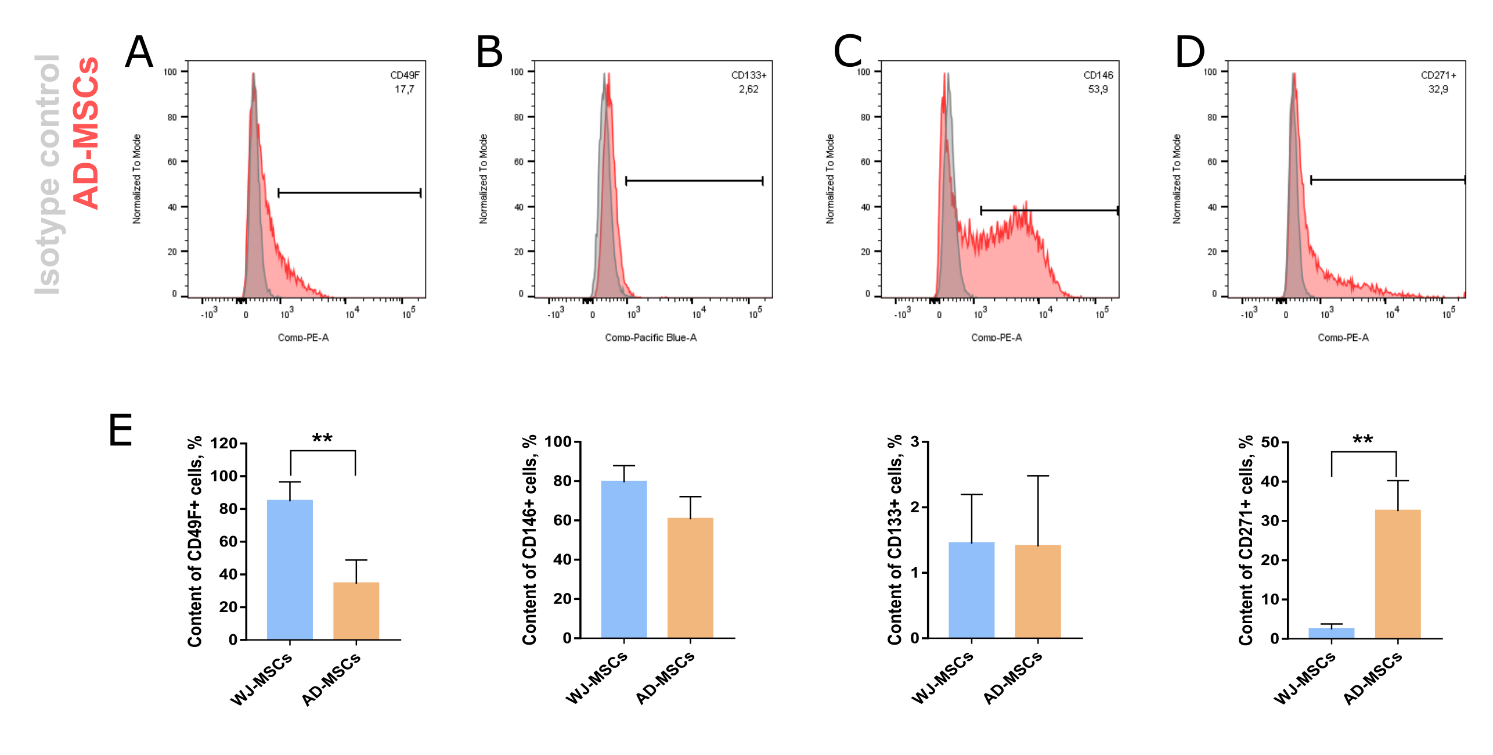


**Supplementary Figure 11**. Expression of surface antigens CD49F (A), CD133 (B), CD146 (C) and CD271 (D) for AD-MSCs; flow cytometry. (E) Expression comparison of CD49F, CD133, CD146 and CD271 between WJ-MSCs and Adipose derived MSC (AD-MSCs). The results are presented as mean values of 3 experiments ± SD. P-value for ** <0.01,


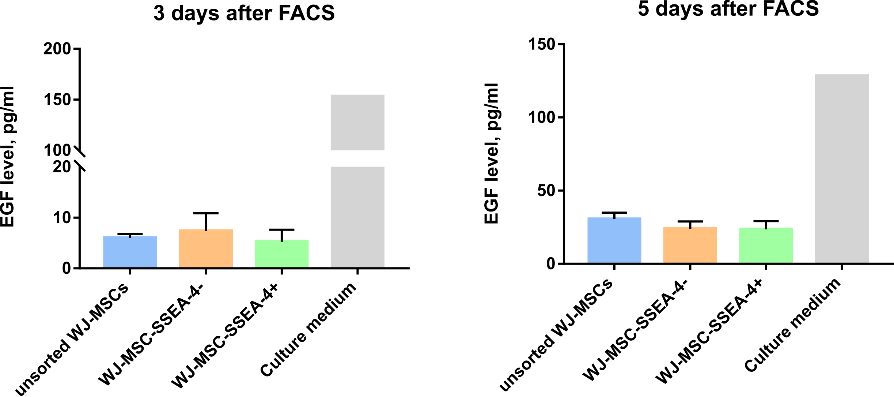


**Supplementary Figure 12**. EGF secretion analysis in 3^rd^ and 5^th^ day in vitro after FACS sorting for unsorted WJ-MSCs, negative population (WJ-MSC-SSEA-4-), positive population (WJ-MSC-SSEA-4+) and in culture medium. EGF levels detected during cell culture were below levels detected in culture medium.
